# Detecting Excess Biofilm Thickness in Microbial Electrolysis Cells by Real‐Time In‐Situ Biofilm Monitoring

**DOI:** 10.1002/bit.29017

**Published:** 2025-05-02

**Authors:** Andreas Netsch, Inka Latussek, Harald Horn, Michael Wagner

**Affiliations:** ^1^ DVGW Research Center, Water Chemistry and Water Technology Karlsruhe Germany; ^2^ Engler‐Bunte‐Institut, Water Chemistry and Water Technology, Karlsruhe Institute of Technology (KIT) Karlsruhe Germany; ^3^ Institute for Biological Interfaces 1 (IBG‐1), Institute for Biological Interfaces (IBG), Karlsruhe Institute of Technology (KIT) Germany

**Keywords:** bioelectrochemical system, biofilm sensor, biofilm thickness, electroactive biofilm, mass‐transfer limitation, microbial electrolysis cell

## Abstract

Long‐term stable operation of bioelectrochemical systems (BES) presupposes the avoidance of mass transfer limitations of the electroactive biofilm. Excessive pH‐gradients from bulk to electrode interface or substrate limitations of the electroactive biofilm are known to diminish the electrical performance of BES. In this study the impact of the morphology of a mixed‐species electroactive biofilm cultivated on the electrical performance of a microbial electrolysis cell (MEC) was investigated to identify the optimal biofilm for real‐life applications in wastewater treatment. Noninvasive monitoring by means of optical coherence tomography and an industrial biofilm sensor allowed for a real‐time evaluation of the morphology of the biofilm. The maximum current density of approximately 3.5 A/m² was found for a mean biofilm thickness in the range of 100–150 µm, beyond which thicker biofilms caused mass transfer limitations. Along with local biofilm detachment a continuous decline in efficiency demonstrates the need for active biofilm control to adjust the biofilm thickness.

## 1 Introduction

Effective mass and charge transfer is crucial for the efficiency of bioelectrochemical systems (BES) (Yang et al. 2021). For the utilization of electroactive bacteria (EAB) as bio‐catalysts for the conversion of waste streams to energy or value‐added chemicals (e.g., hydrogen), their growth and metabolism must not be hampered by extrinsic limitations. In BES, usually, EAB colonize the electrodes developing a conductive biofilm. Substrates providing a carbon source for the EAB (e.g., acetate, glucose) require to diffuse from the bulk phase to the substratum, while equally the products from the bacterial metabolism require to be removed avoiding the development of local accumulations within the biofilm. Insufficient diffusive mass transfer is generally known as a limitation of productive biofilm systems, including BES (Philipp et al. 2024).

In the case of BES issues associated with the mass transfer focus on two major aspects. The impact of substrate supply to the electroactive biofilm on the current production has been extensively investigated by various groups (Cheng and Logan 2011; Ullah and Zeshan 2020). Generally, higher organic loads allow for greater current densities. Substrate‐limiting conditions for different biofilm thicknesses have recently been demonstrated by Pereira et al. 2022. For example, the maximum non‐limited thickness of a mixed‐culture biofilm with a bulk acetate concentration of 8 mmol/L (equivalent to a chemical oxygen demand (COD) = 512 mg/L) was found at a mean biofilm thickness of 55 µm. The second issue that needs to be considered is the accumulation of protons within the electroactive biofilm, leading to local acidic environments. With the conversion of organic substrates, usually protons are produced to maintain charge neutrality in the solution. *Geobacter sulfurreducens*, a model EAB, is completely inhibited at pH < 5, and their growth rate drastically decreases in more acidic environments (Patil et al. 2011). Similarly, the current production by *G. sulfurreducens* was reduced by 50% when the bulk pH is reduced from 6.9 to 6.15, corroborating the inhibition of the metabolism by acidic pH environments (Renslow et al. 2013). In laboratory experiments, pH gradients are often tried to be avoided by the addition of buffer systems to the medium. Too weak buffer systems though result in buffer‐limiting conditions, depending on the thickness of the biofilm (Pereira et al. 2022).

Charge transfer of electrons from the oxidation reaction of the EAB to the electrode is considered the other limiting factor for efficient BES. Electron transfer from EAB to the electrode is commonly described by two different mechanisms of extracellular electron transfer. Direct electron transfer (DET) via cytochromes or conductive transfer via bacterial nanowires and mediated electron transfer via electron shuttles/redox mediators (Schröder 2007). The distance of DET mechanisms is limited. DET via cytochromes, incorporated in the bacterial periplasm require direct physical contact with the electrode, limiting this mechanism to a monolayer of bacteria (Schröder 2007). Conductive pili, however, enable the conduction of electrons to further distant solids (Reguera et al. 2006). Even though considering the electroactive biofilm as a conductive matrix of bacterial nanowires, with increasing distance a higher electrical resistance of the biofilm hampers electron transfer towards the electrode (Babauta et al. 2012; Jain et al. 2011). Mediated electron transfer utilizing molecular electron shuttles (e.g., quinones) underlay the same diffusive mass transport restrictions as substrate supply at larger distances of the microorganism to the electrode. Both electron transfer mechanism seem to be dependent on the local pH in the biofilm (Malvankar et al. 2011; Wu et al. 2016).

In summary, though thicker biofilms contain a higher biomass possibly contributing towards current production, inherently the biofilms growing an impermeable substratum will develop mass transfer gradients limiting the current production of the EAB closest to the electrode. This is in contrast to perfused biofilms (e.g., on carbon veils), where substrate supply can be more uniformly distributed supporting a steady‐state development (Greenman et al. 2020; Ledezma et al. 2012). Various models have been developed to describe the relationship between biofilm thickness and current production in microbial fuel cells, suggesting the need for a regulated biofilm thickness to avoid mass transfer limitation (Kato Marcus et al. 2007; Oliveira et al. 2013; Picioreanu et al. 2010). From experimental investigations different optimal thickness ranges for a *G. sulfurreducens* biofilm (between 30 and 100 µm) for maximum current production have been reported (Pinck et al. 2020; Read et al. 2010; Reguera et al. 2006; Sun et al. 2016). Although, Renslow et al. suggested an electron transfer of EAB to the anode over distances of several 100 µm, even passing inactive zones with dead biomass (Renslow et al. 2013). Most experimental studies or theoretical models presented thus far in the literature discussing the impact of biofilm morphology on the current production of microbial fuel cells have investigated single‐species biofilms. By targeting the application of BES for the energy recovery from waste streams, however, mixed‐culture biofilms will form on the electrodes. In contrast to monocultural biofilms, mixed‐species biofilms are far more complex, as the variety of microorganisms present within the biofilm enables symbiotic as well as competitive interactions between microorganisms. For example, under anaerobic conditions commonly found in the anodic chambers of BES and in the presence of hydrogen (from the cathode of a microbial electrolysis cell (MEC)) a common competitive relationship for electron donors that has recently been investigated is between electrogens and hydrogenotrophic or acetoclastic methanogens (Dzofou Ngoumelah et al. 2024; Georg et al. 2020). Herein, the suppression of methanogens is desired to achieve higher current production and Coulombic yields (Jadhav et al. 2019). As the biofilm morphology is among others subject to the bacterial composition of the consortium for mixed species wastewater biofilms, their relationship between morphology and current production is crucial and yet lacks a comprehensive understanding.

In this study, an electroactive wastewater biofilm was cultivated in meso‐fluidic flow cells and closely monitored by means of optical coherence tomography (OCT), a laboratory technique commonly used in the investigation of electroactive biofilms (Hackbarth et al. 2020; Molenaar et al. 2018; Pereira et al. 2022). Additionally, an industrial heat‐transfer based biofilm sensor was integrated into the flow cells to evaluate its feasibility as an on‐line monitoring tool in larger‐scaled BES, in which laboratory monitoring methods are not suitable (Netsch et al. 2025). Aim of this study was to investigate the correlation between the morphology of the mixed‐species wastewater electroactive biofilm and the current density produced in a MEC to identify an optimal biofilm thickness for maximum current production. Thereby providing a desirable thickness to which electroactive biofilms should be controlled to for a stable and efficient current production in real‐life wastewater BES.

## 2 Materials and Methods

### 2.1 MEC Flow Cell Setup

Mesofluidic flow cells as previously described by Netsch et al. and Hackbarth et al. were operated as single chamber MEC (Hackbarth et al. 2020; Netsch et al. 2025). The anodes were made of a graphite‐polypropylene (C‐PP) compound material (PPG86—Whitecell Eisenhuth GmbH & Co. KG, Osterode, Germany) and integrated along the middle axis into the bottom of the flow cell made from polyoxymethylene (POM). The anodic area was A_anode_ = 1951 mm² (L ×B × H = 101 ×20 ×4 mm³). In each flow cell two stainless steel (Grade—V4A—1.4571) electrodes (A_cathode_ = 1437 mm²) were mounted on the sides above the anode with a vertical distance between anode and cathode of 6 mm. The ratio of cathodic to anodic area was approximately 1.36 to avoid cathodic limiting conditions, possibly interfering with the current production. The flow cell was closed with a polycarbonate (PC) optical window to allow for biofilm imaging by means of OCT along the entire anodic electrode area. On the back of the anode a heat‐transfer biofilm sensor DEPOSENS® (Lagotec GmbH, Magdeburg, Germany) was integrated. The mounting of the sensor to the back of the anode enables the biofilm monitoring by the sensor without interfering with the hydrodynamic conditions in the flow cell or introducing a different substratum material at the point of visualization and electron transfer.

**Figure 1:**
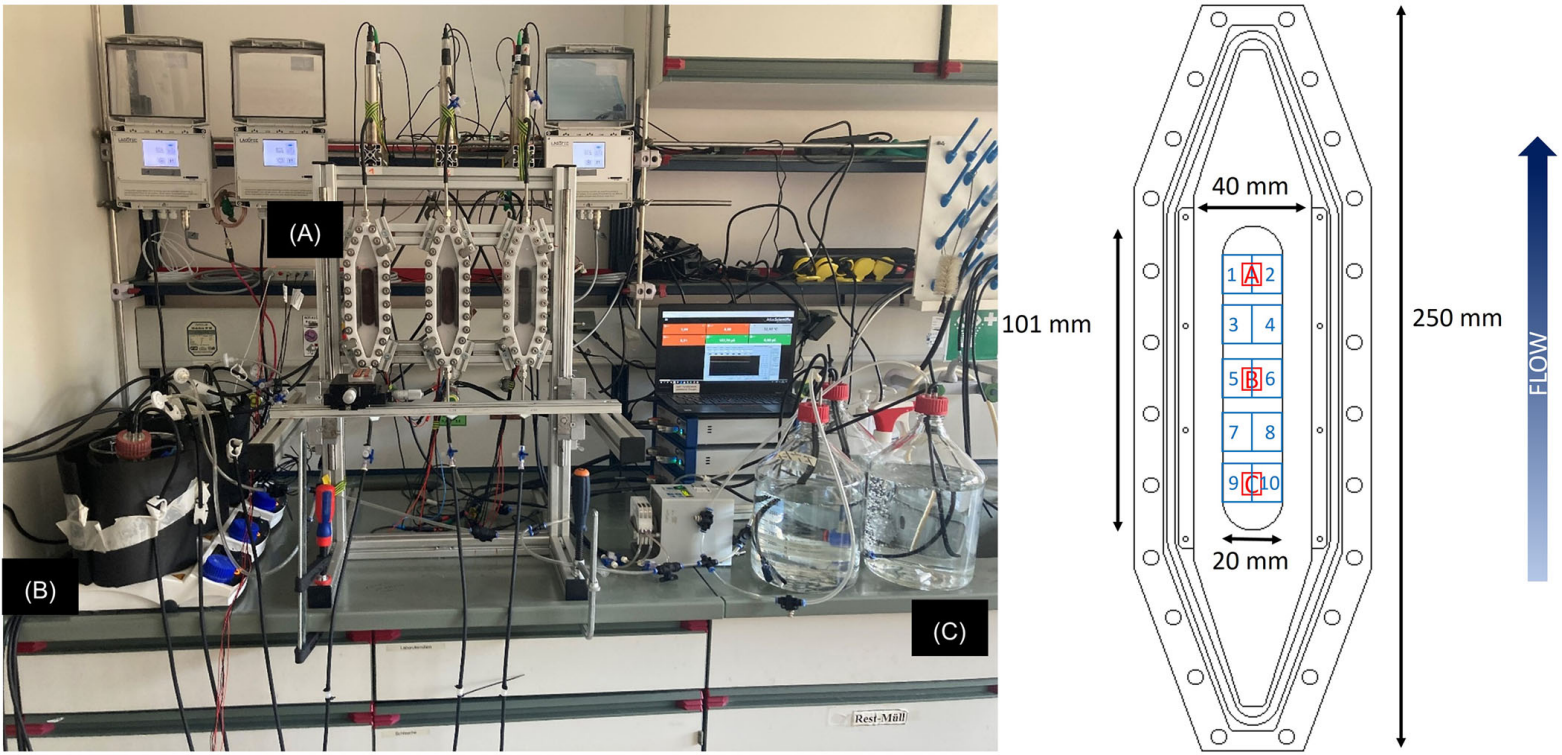
(left) Experimental setup showing three flow cells (A) with integrated biofilm sensor operated in parallel. The cultivation medium was stored in flasks (B) and pumped with magnetic gear pumps (not in image) through the flow cells from bottom to top. Medium for the continuous operation was added into the piping system via peristaltic pump from storage flasks (C). (right) Sketch of the mesofluidic flow cell with the imaging positions for the OCT scans. Red rectangles (4 × 6 mm²) denote the Positions A–C (from top to bottom) that were monitored daily. Blue rectangles (10 × 16 mm²) show the imaging areas (1–10) that were monitored weekly.

Figure 1 shows the experimental set‐up with triplicates of the mesofluidic flow cells connected with flexible tubing (Tygon A‐60‐G, Carl Roth, Germany) to medium flasks and a magnetic gear pump (PAT Niemzik, Haan, Germany) for the recirculation of the cultivation medium. A volumetric flow rate of 100 mL/min was set resulting in a mean flow velocity in the flow cell of 0.5 cm/s. The dimensions of the flow channel were L × B × H = 220 × 40 × 9 mm^3^. In the flasks the medium was continuously sparged with nitrogen gas to ensure anaerobic conditions. The medium was heated to a temperature of 30°C ± 2°C (measured by the medium temperature probe of the biofilm sensor), to maintain constant cultivation conditions.

### 2.2 Biofilm Cultivation Procedure and Medium Composition

The flow cell triplicates were inoculated with a pre‐conditioned wastewater from a microbial fuel cell (MFC) reactor (construction based on (Haupt et al. 2022)) operated for over half a year with an acetate‐based cultivation medium. The inoculum was extracted from the bulk phase of the MFC reactor and anaerobized before introduction to the system. Each flow cell was inoculated with 1 L. After inoculation concentrated cultivation medium was injected into the system. The medium composition was modified from Hackbarth et al. (2023) containing: 84 mg/L KH_2_PO_4_, 44 mg/L K_2_HPO_4_, 40 mg/L NH_4_Cl, 42 mg/L MgCl_2_, 0.2 ml 0.4 mM CaCl_2_ solution, 2 mL nutrient broth trace element solution (100x concentrated) (Coppi et al. 2001), 0.2 mL selenite–tungstate solution (13 mM NaOH, 17 µM Na_2_SeO_3_, and 12 µM Na_2_WO_4_), 2 mL Wolin's vitamin solution (German Type Culture Collection DSMZ 141). The cultivation medium was autoclaved and anaerobized by N_2_‐sparging before introduction to the system. As carbon source sodium acetate (15 mmol/L unless noted otherwise; COD = 960 mg/L) was added.

Biofilm cultivation was performed as batch mode for the first 7 days of the experiment to avoid flushing out planktonic bacteria and support initial attachment of the biofilm. After 7 days the medium was continuously replenished with cultivation medium at a volumetric flow rate of 0.4 mL/min with a peristaltic pump (Reglo digital, Ismatec). Excess medium from the system was removed via overflow. Since, the focus of this study was the cultivation of thick anodic biofilms causing mass transfer limitations in the close proximity of the anode at a constant medium composition. The reactor medium was continuous replenished aiming to set a constant acetate concentration and buffer capacity. Thereby, removing the effect of varying medium composition observed in batch or fed‐batch experiments.

Two experimental runs were performed with each triplicate flow cells, however in experiment B in one flow cell no electroactive biofilm was cultivated successfully. Table 1 shows the conditions for the experimental runs. Note that the system volume was reduced in Exp. B due to the change of 1 L flasks to 0.5 L flask for the integration of dissolved oxygen probes.

**Table 1:** Overview of the experimental conditions of both experimental runs. Note that in Exp. B in one flow cell no electroactive biofilm developed and was therefore excluded from the study.

	Duration (d)	No. flow cells	V_system_ (L)	c_acetate_ (mmol/L)	COD (mg/L)	OD_600, Inoculum_
Exp A	54	3/3	1.3	20	1200	0.1
Exp B	62	2/3	0.8	15	960	0.05

### 2.3 Sampling and Measurements

Liquid samples (5 mL) were taken from the bulk medium and the inflow daily. Acetate concentrations were determined via ion chromatography (Metrohm 881 Compact Pro Ion Exchange Chromatograph with a Metrosep Organic Acids 250/7.8 column, Metrohm, Switzerland). Electrical conductivity and pH of the liquid samples were measured daily with lab‐grade probes (WTW—SenTix 41, TetraCon 325, Xylem, Weilheim, Germany). Dissolved oxygen concentration was monitored with fiber‐optical oxygen probes (ROB10, Pyroscience, Aachen, Germany). The optical cell density (OD_600_) was measured with a UV/VIS spectrometer (Lambda XLS +, PerkinElmer, Rodgau, Germany).

### 2.4 Biofilm Monitoring and OCT Analysis

Biofilm development on the anode of the MEC was monitored by means of OCT and the heat‐transfer biofilm sensor. The biofilm sensor measures the increasing thermal resistance on the substratum due to accumulating biofilm and reports a dimensionless signal (in arbitrary units a.u.) as output. For the validation of the sensor signal as indicator for the biofilm thickness it was correlated with the biofilm thickness calculated from OCT images at the position of the sensor (OCT Position A—see Figure 1 (right)). A more detailed description of the working principle of the biofilm sensor has extensively been describe in a previous publication by this group (Netsch et al. 2021, 2025). The temperature difference between heater and medium probe was set at Δ*T* = 5 K to reduce impact of locally different cultivation temperature. The sensor's reference measurement in clean state was set to zero at a volumetric flow rate of 100 mL/min.

Additionally, for validation of the sensor measurement and in‐depth analysis of the morphology of the electroactive biofilm daily OCT‐scans of three selected positions A–C (W × L = 4 × 6 mm²) along the middle axis of the anode were performed monitoring a total surface of 72 mm² (approximately 4% of total anodic area). OCT images were acquired with a GANYMEDE spectral‐domain base‐unit (GAN610, Thorlabs GmbH, Lübeck, Germany) with a OCT9G scanner and OCT‐LK4‐BB lens (all components from Thorlabs GmbH, Lübeck, Germany). The lateral pixel resolution was set to 8 µm/px and vertical pixel resolution to 2.06 µm/px. As the entire biofilm growing on the electrode contributes towards the anodic current generation a “full scan” of 10 positions over the entire width of the electrode (W × L = 10 ×16 mm²) were taken weekly to visualize the biofilm on a total of 82% of the anodic area. Images of the “full scan” reveal the representativeness of the daily OCT imaging scheme and uncover uneven biofilm distribution. To reduce the data quantity the “full scan” images were taken with a reduced lateral pixel resolution of 24 µm/px. Imaging positions on the electrode are displayed in Figure 1.

Biofilm parameters obtained from the OCT images were calculated according to Wagner and Horn using in‐house ImageJ macros (Wagner and Horn 2017). The distance of the bulk‐biofilm interface to the electrode is described by the mean biofilm thickness ${\overset{-}{L}}_{F}$ with $L_{F,i}$ being the local biofilm height (Equation 1). The substratum coverage (SC) specifies the percentage of electrode, on which biofilm has grown (Equation 2). OCT images are displayed in the later sections as height maps showing the distance of the bulk‐biofilm interface from the substratum (electrode).

(1) $${\bar{L}}_{F}=\frac{1}{N}\cdot \sum_{i=1}^{N}L_{F,i}\;\left(µm\right)$$

(2) $$\mathrm{SC}=\frac{A_{\mathrm{OCT}}-A_{\mathrm{uncovered}}}{A_{\mathrm{OCT}}}\;(\%)$$

### 2.5 Electrochemical Measurements

Anodes (working electrode) were contacted directly by a socket incorporated into the anode material, while the cathodes (counter electrode) were contacted with Grade 2 titanium screws. An Ag/AgCl‐reference electrode (SE23‐I, Xylem Analytics, Waldheim, Germany) was installed at the outlet of the flow cell in a custom‐made stainless‐steel electrode holder. All electrodes were connected with a potentiostat (Interface 5000 P, Gamry Instruments, Warmister, USA) for anodic potential control and electrochemical measurements. Calculated current densities from the chronoamperometry are in reference to the anodic area A_anode_ = 1951 mm². The Coulombic Efficiency (CE) was calculated according to Equation 3, giving the ratio of electroactively consumed acetate to the total acetate consumption in the system. The CE was determined for each time interval between two liquid sampling points (*t*_2_ and *t*_1_), whereas ∆c(acetate) described the total acetate consumption in the time interval. The total amount of electroactively consumed acetate was determined by the number of electrons released to the anode. This was calculated by the integral of the resulting current I in the time interval, the number of electrons released per mol of acetate (z_e_ = 8), the reactor volume (*V*) and Faraday's constant (*F* = 96485 C/mol).

(3) $$\text{CE}=\frac{\int_{t1}^{t2}I\left(t\right)\text{dt}}{z_{e}\cdot V\cdot ∆c\left(\text{acetate}\right)\cdot F}\;\left(\%\right)$$

## 3 Results

### 3.1 Current Development

**Figure 2:**
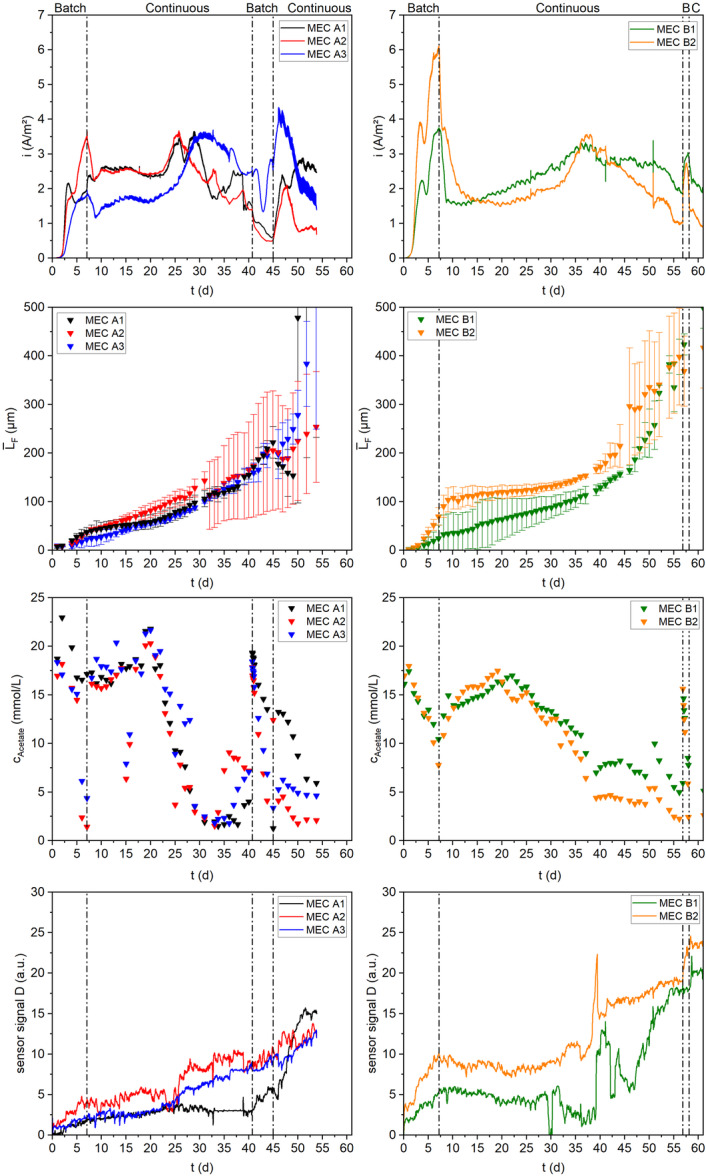
From top to bottom the development of the current density, mean biofilm thickness, concentrate of acetate in the bulk medium and sensor signal shown for the MECs of experiment A (left) and B (right). Compare with Table 1.

Figure 2 shows the development of key parameters (current density *i*_anode_, mean biofilm thickness ${\bar{L}}_{F}$, bulk concentration of acetate *c*_acetate_ and sensor signal *D*) in the MECs over the course of the cultivation period. Anodic current production commenced within the first 2 days after inoculation and displayed a rapid development along with increasing mean biofilm thickness. Interestingly, between Days 3 and 4 of the experiment the current production in four of the MECs seems to stagnate (A2) or even decrease (A1, B1, and B2), before continuing the rapid trend of current increase (in detail see Figure SI‐1). Metabolic changes of the EAB from catabolism to anabolism on the anode may explain the inflection in the current development. This effect was possibly not visible for MEC A3 due to an overlap of the exponential growth phase on different sections of the anode due to the delayed bacterial growth.

After 7 days of cultivation, the MEC operation was switched from batch mode to continuous addition of fresh medium and removal of bulk medium. Before changing the operation conditions all MECs reached a maximum current density of 2.4 A/m² (A1), 3.5 A/m² (A2), 1.8 A/m² (A3), 3.7 A/m² (B1) and 6 A/m² (B2). While the steep increase of the current density was aligned with the rapid anodic biofilm development the height of the current density peak must be put in relation to the pH of the medium. During batch operation the pH increased from 7.2 at inoculation to a pH between 9.0 and 9.2 for all flow cells. The drop of the current density with the continuous addition of medium (pH = 7.2), therefore, is likely the consequence of the resulting pH decreases to more neutral values around 7.5.

During continuous operation the current density remained stable for approximately 10 days in the MECs A1, A2, and B2. While MECs A3 and B1 still showed an increasing current density presumably along with the delayed biofilm growth on some parts of the electrode (compare Section 3.2 After approximately 25 days (A1 and A2) or 30–35 days (A3, B1, and B2), respectively, all MECs reached a secondary maximum current density at approximately 3.5 A/m². Beyond the maximum the MECs deteriorated in their stability and total current output. Despite, the continuous replenishment of the cultivation medium, the acetate concentration in all MECs steadily decreased along with the current density. Simultaneously, these MECs showed an increased consumption of acetate by non‐electroactive bacteria in the system, which in summary leads to a reduced CE, from approximately 40%–60% in the early stages of the experiment to 5%–15% during the latter stages (more details in Figure 6). The decreasing current production after the secondary maximum may have been caused by a reduced concentration of acetate in the bulk, that could have led to a substrate limitation of the EAB in the proximity of the anode.

To determine, if the anodic biofilm was substrate‐limited at Days 40 (Exp A) and Day 57 (Exp B) continuous medium addition was interrupted and the acetate concentration was increased to 20 mmol/L (COD = 1200 mg/L) or 15 mmol/L (COD = 960 mg/L), respectively. In Exp B in both MECs the current rapidly increased to a range similar the secondary maximum, indicating the limiting factor for current production was the availability of the carbon source for the electroactive biofilm. In contrary, the MECs of Exp A showed an additional drop in the current density suggesting other limiting factors beyond the substrate availability reduced the current production.

### 3.2 Biofilm Development and Detachment

**Figure 3:**
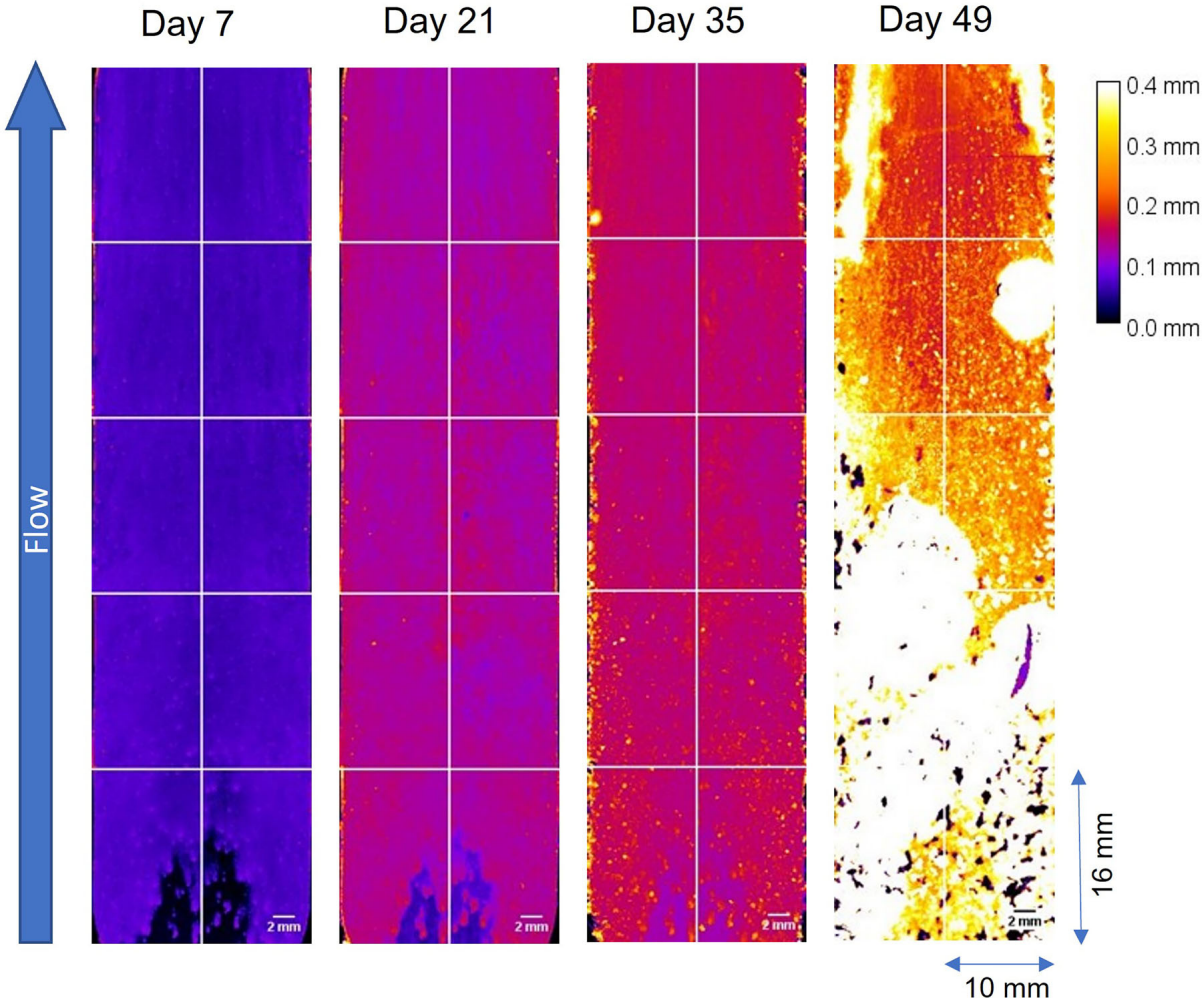
Height maps derived from OCT images showing the distance of the bulk‐biofilm interface from the electrode substratum of MEC B2 (see Table 1) For Days 7, 21, 35, and 49 a full scan displaying approximately 82% of the electrode were taken. The height map displays the thickness of the biofilm according to the heat map for the range of 0–400 µm. The direction of flow was from bottom to top.

The electrodes of MECs A1, A2, and B2 showed an almost complete coverage of the substratum within the first few days after inoculation. As an example, Figure 3 shows the height maps deriving from OCT images of a “full scan” of the electrode of MEC B2. The height maps of the other MECs are additionally provided in the Supporting Information. The biofilm in all MECs grew mostly steady with a growth rate between 3 and 7 µm/d until approximately Day 35. The distribution of the biofilm was mostly homogeneous for the electrode areas that were well covered during the inoculation, visible by the comparatively low standard deviation of the mean biofilm thickness for MECs A1, A2, and B2 (see Figure 2). In contrary the electrode of MECs A3 and B1 was unevenly covered, whereas larger areas of the electrode (imaging position 1—closest to the inflow) initially remained uncovered (compare Figure SI‐2). Possibly, the free jet at the inlet has caused increased shear forces hampering bacterial attachment (compare with fluid dynamic simulation by Hackbarth et al. (2020)). This is in line with the delayed coverage of the electrode found by Godain et al. (2024) when cultivating an electroactive biofilm at higher shear stress. The uncovered areas however, were covered within the next few days, so that biofilm growth simply was delayed. This aligned with the reduced current production in both MECs.

**Figure 4:**
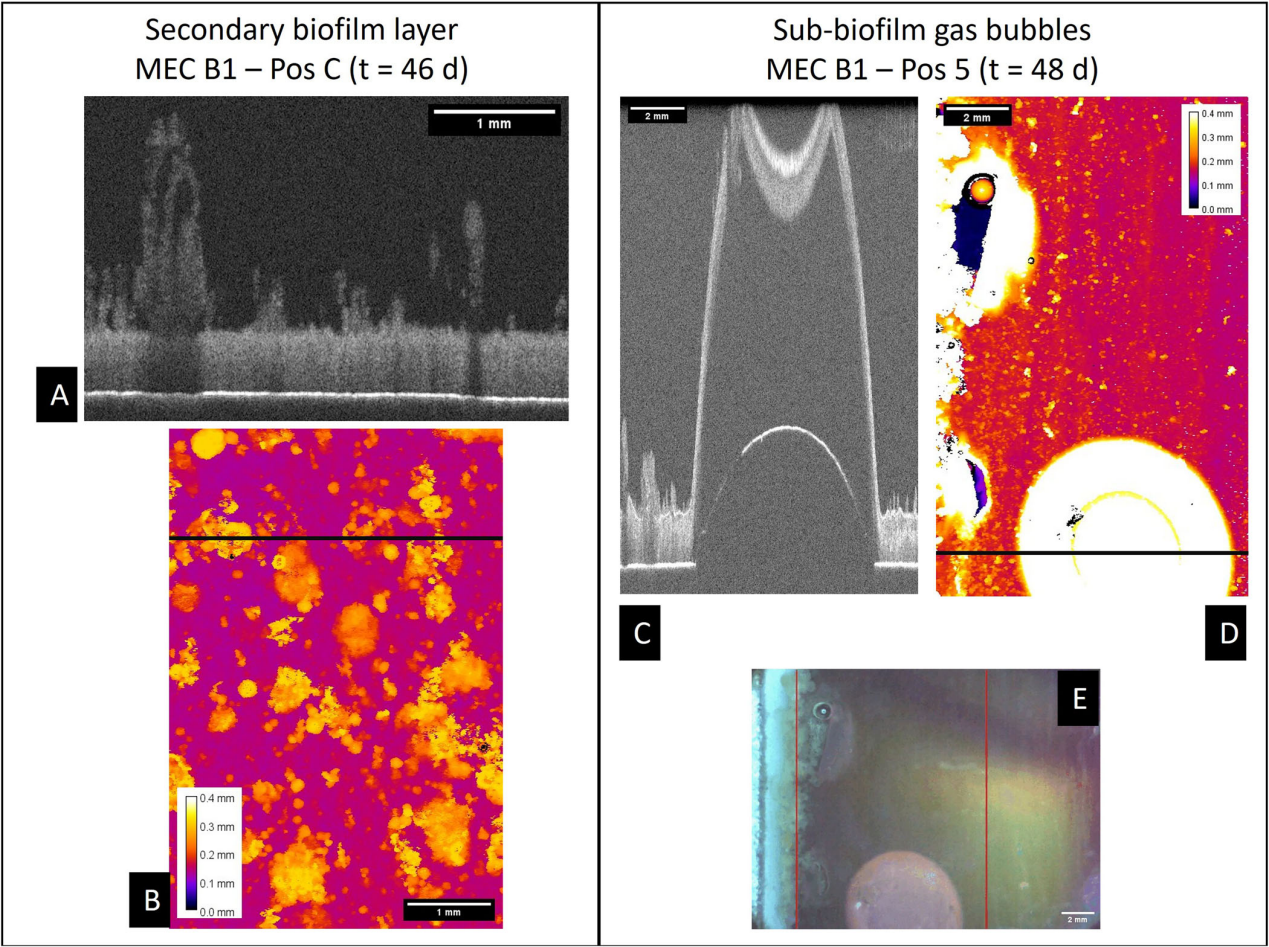
(left) Shows the growth of fluffy high structures of the secondary biofilm layer, that developed as patches on top of the homogeneous primary biofilm layer. A cross‐section (A) and the corresponding height map (B) are displayed. The high yellowish patches in the height map display areas covered by the secondary biofilm layer. (right) Shows a gas bubble entrapped below the biofilm in MEC B1 on Day 48. Displayed are a cross‐section (C), the height map (D) and the corresponding image (E) of the biofilm. The black lines in the height maps mark the position of the respective cross‐sections.

After Days 30–35 in both experiments, OCT imaging detected a rapid increase in the biofilm accumulation rate, accompanied by a rising sensor signal from the biofilm sensor. The surge of the biofilm accumulation rate can be explained with two different causes. Firstly, it seems a secondary biofilm layer rapidly developed on top of the initial biofilm layer (compare Figure 4A,B). While the bottom layer displayed a very homogeneous and compact structure (reddish color) the secondary biofilm grew spottier in higher filamentous patches (blackish/yellowish). The different morphologies of the two biofilm layers indicate the dominance of different bacteria in the two respective biofilm layers. We infer that the slow growing bottom biofilm layer was dominated by EAB contributing towards the current production, while the secondary biofilm layer likely originated from another fast‐growing microbial species.

The second cause for the biofilm deterioration was a partial detachment of the biofilm mainly caused by gas bubbles (most likely CO_2_, or CH_4_) forming at the anodic interface. An example of this phenomenon is displayed in Figure 4C–E, where over several days, a locally isolated gas bubble developed below the biofilm surface with an approximate area of 12.5 mm² (~0.5% of the anode area). Consequently, the electroactive biofilm layer was locally lifted from the anode and partially ripping patches of biofilm off. The structure of the biofilm seemed to prevent the diffusion of gaseous products from the conversion of organic substrates at the anode. Biofilm detachment events were visible on several occasions, although the biofilm was rarely completely detached. Below the lifted biofilm, the electrode was freed from biofilm, suggesting the gas bubbles formed directly at the electrode interface. However, the detachment events could not be directly correlated with a sudden drop of the current density, but are in line with the overall decreasing current density beyond approximately Days 30–35, due to the limited area affected by the individual detachment events and the relatively long time interval (several days) for the development of the gas bubbles. For example, the detachment event in MEC B1 on Day 48 (compare Figure 2) did not show a change in the decreasing trend of the current density. Though not clearly distinguishable, it is suggested that both causes may have contributed towards the deteriorating current production as a result of the biofilm structure.

### 3.3 Correlation Biofilm Thickness and Current Density

**Figure 5:**
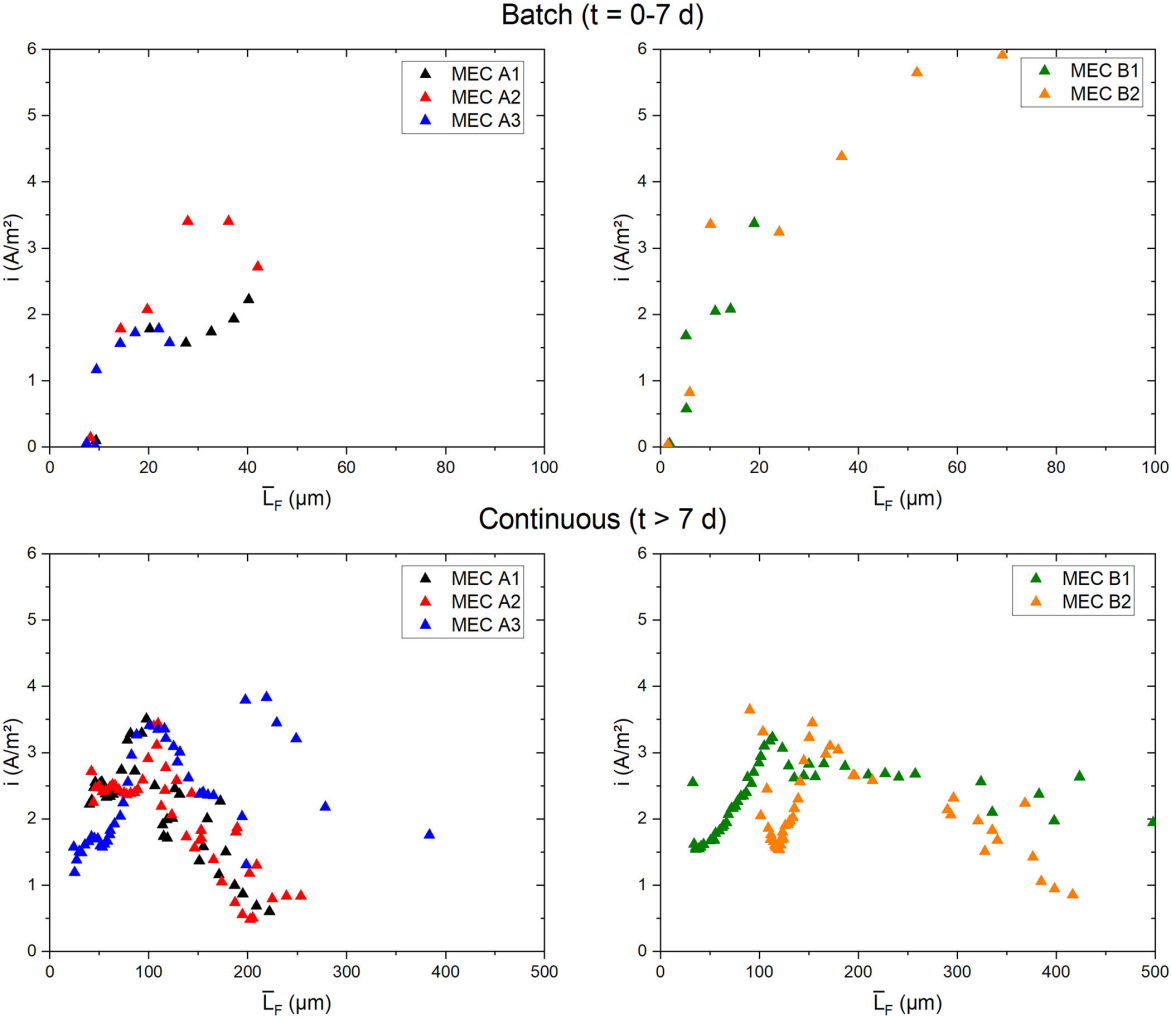
Correlation of the current density with the mean biofilm thickness (Pos. A–C—compare—Figure 1) of the initial growth during the batch phase (Day 0–7—top) and during continuous operation *t* > Day 7 (bottom). Note the different scale of the *x*‐axis for the top and bottom graphs.

The dependency of the current density from the accumulated biofilm on the electrode is displayed in Figure 5, in the top subplots separately for the first 7 days during batch mode and the correlated data points during continuous operation (bottom subplots).

During the initial attachment and growth phase of the electroactive biofilm in batch operation of the first 7 days a steeper correlation of the current density with the mean biofilm thickness was visible, compared to the growth trends during continuous operation up to the maximum current production. The slope of this correlation during batch operation (97.08 ± 32.84 A/mm³) exceeds the slope of the current growth up to its maximum during continuous operation (26.29 ± 12.9 A/mm³) by a factor of 3.7 (compare Figure SI‐7 and Table SI‐1). This decrease in the specific current production per volume of biomass indicates a decreasing efficiency of the electroactive biofilm growing at larger distances from the anode. Hereby, two factors may simultaneously play a role, as with increasing distance from the electrode an increased conductive resistance of the biofilm matrix has been found (Malvankar et al. 2012). On the other hand, the larger substrate gradient may have decreased the efficiency of the electroactive bacteria in the closer proximity of the anode.

All MECs reached a maximum in their produced current density of between 3.2 and 3.5 A/m² at a mean biofilm thickness of 100–115 µm. Although MEC B2 having an increased biofilm growth, it reached its maximum at a mean biofilm thickness of 150 µm. Further biofilm growth however, did not contribute towards an elevated current production of the MECs (see Figure 5). A steady decrease of the current density can be seen for all MECs at higher biofilms thicknesses. This may be a consequence of the increasing diffusion depth for both educts (acetate) and products (protons and CO_2_) of the metabolism of the EAB. Interestingly, the decline of the current density differed in the magnitude among the MECs. While MECs A1 and A2 produced only a current density of less than 1 A/m² at a mean biofilm thickness in the range of 200 µm, the MECs A3, B1, and B2 were able to produce approximately 2 A/m² with a mean anodic biofilm thickness between 300 and 400 µm.

**Figure 6:**
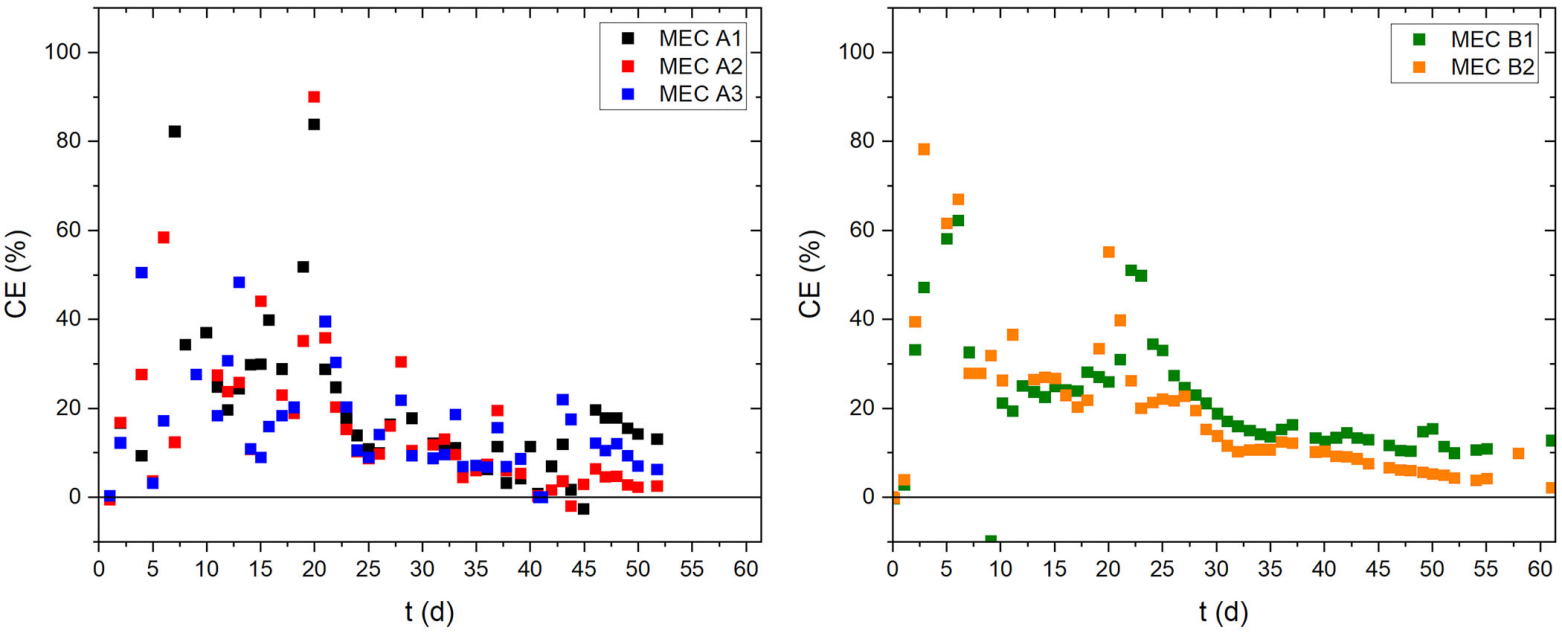
Development of the coulombic efficiency (CE) of the MECs over the course of the biofilm cultivation. The CE was calculated based on Equation 3 for the time interval between to sampling points of 24 h.

The decline in the efficiency of the electroactive biofilm could also be seen in the development of the CE over time. Figure 6 displays the CE for all MECs of time.

The highest efficiency in terms of fraction of acetate consumption by the EAB was visible towards the end of the initial growth phase in batch mode (Day 7). Here, due to the polarization of the anode at −199 mV vs. Ag/AgCl, a favorable niche supports the high selectivity for EAB. Similar to the current development, a lower secondary maximum of the CE was visible, that coincided with the peak acetate concentration during the continuous mode. This secondary maximum was in the range of 35%–50%. Interestingly, the maximum point, around Day 20 for all MECs, preceded that of the current density by a few days. Possibly, the secondary growth phase during the continuous mode may have been triggered by an increased acetate concentration, leading to the higher CE. For the remainder of the biofilm cultivation; however, a continuous decline of the CE was visible. This aligns with the suggested increased acetate consumption by non‐electroactive bacteria. Between Days 45–50 the CE for all MECs had decreased to 5%–15%.

### 3.4 Evaluation of State of Anodic Biofilm by Means of the Biofilm Sensor

**Figure 7:**
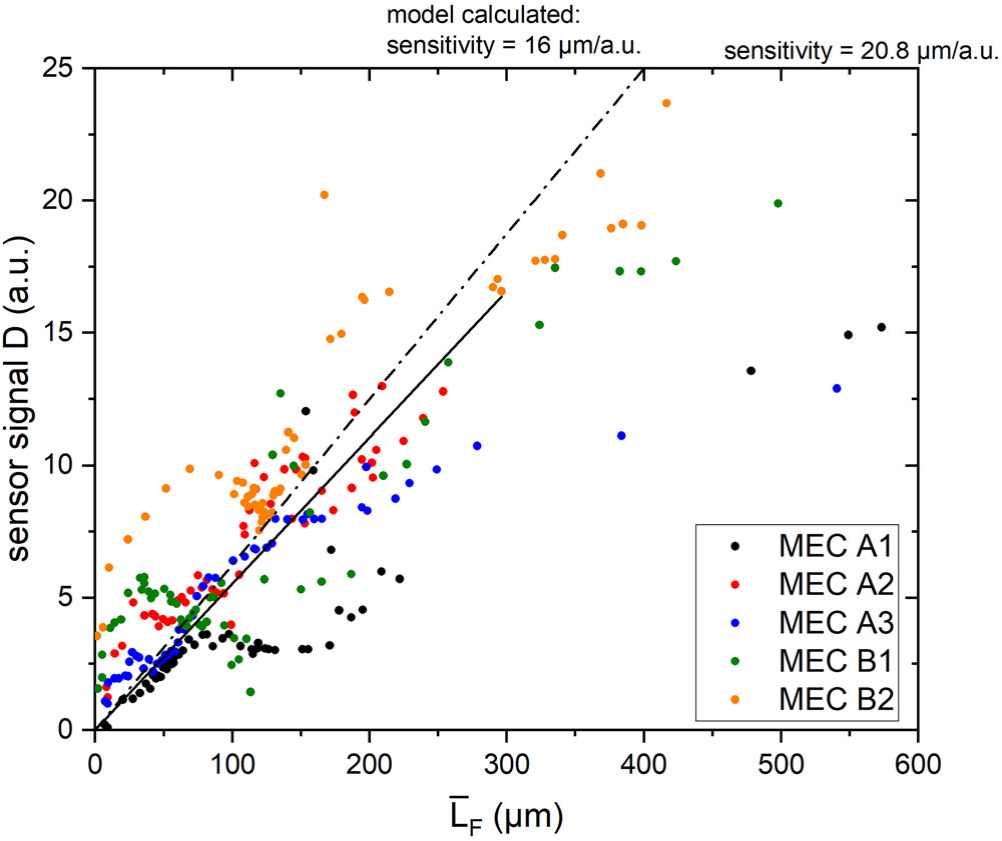
Correlation of the dimensionless sensor signal with the mean biofilm thickness calculated from the OCT scans of Pos. A (compare Figure 1).

From the correlation of the current density with the mean biofilm thickness on the anode, an optimal biofilm thickness range for maximum current production can be deduced for continuous operation. This suggests for long‐term stable operation the control of the biofilm thickness within the range of 100–150 µm. Biofilm monitoring of the anodes in the mesofluidic flow cells was performed by the installed heat‐transfer biofilm sensor on the back of the anode and confirmed by means of OCT scans. The correlation of the biofilm sensor signal with the mean biofilm thickness at the OCT Position A (point of sensor measurement) is displayed in Figure 7.

Despite subject to some fluctuations the sensor signal and mean biofilm thickness displayed a linear correlation (*R²* = 0.87) with a resulting sensitivity of 20.8 µm/a.u. Deviations from the linear correlation may have been caused by unstable flow conditions due to excessive biofilm growth in the periphery of the flow cell at later stages of biofilm cultivation. Rapid increases/decreases in the sensor signal are caused by a variance of the volumetric flow rate, then during zeroing at the beginning of the experiment (compare Figure 2). Additionally, if the minimum required flow velocity for a sensor measurement by means of the biofilm sensor was not exceeded, the sensor signal may have stagnated, for example, MEC A1. Applying the model for the calculation of the sensor sensitivity presented in Netsch et al. 2025 with the volumetric flow rate of 100 mL/min an improved sensitivity of 16 µm/a.u. is suggested compared to the determined sensitivity in this study. The deviation may be explained with the nonlinearity of the sensor sensitivity for thicker biofilms (Netsch et al. 2025).

As mentioned above, the sensor signal was correlated with the OCT‐scans of Pos. A in the flow cells imaging the point of sensor measurement. However, since the biofilm on the entire surface of the electrode contributes towards current production, the representativeness of the biofilm measurement needs to be investigated. Comparing the mean biofilm thickness at Pos. A with the mean biofilm thickness derived from full scans including approximately 80% of the electrode area revealed a slight underestimation. With the exception of MEC B1, the biofilm at Pos. A generally had the tendency to be thinner by a margin of up to approximately 25% compared to the full scan within the first 41 Days of the experiment. The non‐equal biofilm growth was likely a consequence of local variations in the hydrodynamic conditions, as also found by Hackbarth et al. using the same flow cell design (Hackbarth et al. 2020). Later comparisons were subject to larger fluctuations due to local detachment events or spotty overgrowth, which increasingly compromised the representativeness of the OCT images. A more detailed analysis is shown in the Supporting Information (SI). For upscaled BES with larger electrode areas, the uneven biofilm distribution displayed here, would suggest the use of multiple sensors to avoid misinterpretation due to nonhomogeneous biofilm growth or locally limited detachment events, in consideration of the hydrodynamic conditions of the reactor.

## 4 Discussion

As previously described electroactive biofilms are influenced by the trade‐off between high bacterial accumulation in the biofilm and the resulting mass transfer limitations from the biofilm morphology. Diffusion coefficients of dissolved species in biofilms are reduced by several biofilm characteristics, including high cell density (Fan et al. 1990), low porosity (Yang et al. 2021), high biofilm age and large distance from the bulk phase (Renslow et al. 2010). Furthermore, Renslow et al. (2010) found a large variability of the local diffusion coefficients in the interior of biofilms due to the local variability of the biofilm structure. The development of sub‐biofilm gas bubbles (compare Figure 4) may hint towards local insufficient diffusive mass transfer of reaction products. Comparing the diffusion coefficients of the possible gases CO_2_ and CH_4_ with that of the substrate (acetate) it becomes obvious, that diffusion restrictions affecting the transport of product gases out of the biofilm will similarly impede the diffusive transport of substrate into the biofilm, as the diffusion coefficient of acetate is slightly lower than those of CO_2_ and CH_4_ (compare Table 2).

**Table 2:** Comparison of the diffusion coefficients in water for the relevant chemical species in the electroactive biofilm. The diffusion coefficients were taken from Cussler (2009).

Chemical species	Diffusion coefficient in water (× 10^−6^ cm²/s)
H^+^	93.1
CO_2_	19.2
CH_4_	14.9
Acetate	12.1

Reduced diffusion coefficients along with large biofilm thickness lead to mass transfer limitations in the electroactive biofilm resulting in substrate limitations (Pereira et al. 2022), as this was the case in the MECs of Exp B, visible during the spiking with substrate in the batch experiment. The substrate limitation in this study may has been enhanced by the development of the fast‐growing non‐electroactive secondary biofilm layer (Sleutels et al. 2016). The increased diffusion length for the substrate along with the increased substrate consumption of the secondary layer, has reduced the availability of substrate for electroactive consumption in the proximity of the anode, reducing not only the CE, but also the total current production.

Also, more acidic pH environments in the anodic proximity (Li et al. 2017; Renslow et al. 2013) as a consequence of increased diffusion length can lead to a reduced electroactive metabolism. Franks et al. (2009) showed the magnitude of the resulting pH gradient in a *G. sulfurreducens* biofilm, with a decrease of 1 pH‐unit in an approximate 70 µm thick biofilm. Although the slope of the pH‐gradient depends on the respective biofilm characteristics, the 200–400 µm thick anodic biofilms in this study likely have developed an even larger pH decrease from bulk towards the anodic interface. The effect of the pH on EAB has been demonstrated by Patil et al. (2011), showing the highest current production at a pH around 9, while in acidic pH environments pH < 5 EAB are partially or completely inhibited, which is further reinforced by weak‐buffer systems (Pereira et al. 2022). Metabolically inactive regions will result in less effective biofilms. Both substrate limitation and pH inhibition occur simultaneously in a BES, as they will similarly increase with growing biofilm thicknesses and might not be distinguishable in the decline of the produced current.

Yang et al. (2021) suggest the regulation of the biofilm thickness and porosity to enhance mass transfer in an electroactive biofilm. This poses the question of an optimal electroactive biofilm thickness in BES. This may simplify the effect of the biofilm morphology in the mass transfer; however, the monitoring of the continuous growth by means of the biofilm sensor might be an easy and viable indicator for the prediction of impaired current production due to the anodic biofilm morphology. The optimal biofilm thickness for current maximum production has mostly been investigated for mono‐cultural biofilms (e.g., *G. sulfurreducens*) showing viable biofilms up to a thickness of 80 µm (Pinck et al. 2020). Considering, however, the application of microbial fuel cells in wastewater treatment, a more complex mix of microbial species is expected. Similar to the overgrowth of the electroactive biofilm layer in this study (compare Figure 4A,B) undesired microorganism in the electroactive biofilm should be removed or their growth suppressed. Such an overgrowth of an electroactive biofilm was also reported by Yan et al. (2021) in a *Geobacter* dominated biofilm, where aerobes seemed to have developed a secondary biofilm layer, that could be removed by washing with norspermidine and recovering the *Geobacter* dominance of the biofilm.

In this study a complex mixed culture preconditioned from a municipal wastewater treatment plant was used, developing the maximum current production during continuous operation a higher range of thickness between 100 and 150 µm. If the described optimal range of biofilm thickness found in this study can be applied widely for different mixed culture electroactive biofilms, requires more extensive analysis. Though, a global study by Santoro et al. (2021) found comparable power outputs from different wastewater inocula in identical systems, while the enriched bacteria in the biofilm differed from each other.

Although not specifically identified, large‐scale pilot‐plant microbial fuel cells in wastewater treatment applications by several groups (e.g., Blatter et al. (2021) or Babanova et al. (2020)) have shown a continuous decline in the current production, possibly attributed to increasing mass transfer limitations. The decline in current production and CE over time along with increasing anodic biofilm thickness in this study, clearly shows the necessity of biofilm control mechanisms. Unlike biofilm control in technical systems such as, for example, membrane applications a complete biofilm removal is not targeted. Moreover, given the maximum current production found in the range of 100–150 µm the aim should be to continuously set the biofilm to this thickness. It was shown by Islam et al. that periodic treatment ultrasound of an MFC with carbon brush electrodes revitalized the electroactive biofilm to the same maximum current density as before its decline over a period of 40 days (Islam et al. 2017). Similarly, flow‐induced increase of shear forces was investigated by the same group of researchers showing a recovery of the current production (Islam et al. 2019). In both cases, the physical control mechanisms removed large fractions of the biofilm followed by a quick reattachment of living cell. Thereby, showing the need for a removal of dead cells, as a high ratio of living cells in a thinner biofilm is most desirable (Islam et al. 2019). Heat‐transfer biofilm sensors can allow for a condition‐based use of control mechanisms, by detecting excess biofilm thickness or surges in the biofilm growth rate which might indicate an overgrowth of the electroactive biofilm layer. Applicable biofilm control mechanisms may be limited due to constructive restrictions in BES reactor design. Control mechanisms used in other fields of productive biofilm applications may present more viable options. Hwang et al. used nitrogen sparging to adjust the biofilm thickness in a membrane biofilm reactor with a nitrifying biofilm (Hwang et al. 2010). Wei et al. (2024) used air‐scouring in a membrane aerated biofilm reactor for biofilm control, while Zhang et al. (2017) used mechanical abrasion to remove salt precipitation and biofilm from an air cathode in microbial fuel cells. Although, targeting different deposits on the substratum the control mechanisms were equally utilized to remove mass transfer limitations in productive biofilm applications.

## 5 Conclusions

Within this study, the development of the anodic biofilm in a MEC was monitored over a period of up to 62 days to quantify the relationship between the accumulation of biofilm and current production. A trade‐off between the accumulation biomass and the cultivation niche for electroactive bacteria within the microbial consortium, driven by the availability of electron donor (nutrients) and electron acceptor (anode) is suggested for the mixed‐species wastewater biofilm.

- The optimal range of biofilm thickness for an electroactive biofilm cultivated from wastewater was determined between 100 and 150 µm producing up to 3.5 A/m².
- A combination of mass transfer limitation due to the excess biofilm thickness along with increased substrate consumption by non‐electroactive bacteria have led to decreasing substrate concentration in the proximity of the anode. The lower availability of substrate for EAB resulted in a declining current production.
- The deteriorating biofilm performance was caused by a combination of overgrowth of the primary electroactive biofilm layer with a fast‐growing secondary biofilm, along with local detachment of the primary biofilm due to gas development beneath the biofilm at the anodic interface.
- To identify the optimal biofilm thickness in large‐scale applications, heat‐transfer biofilm sensors can viably support the monitoring of electroactive biofilm in the electrodes of BES. However, for the evaluation of the performance of the BES, the sensor information must be coupled with knowledge of the local hydrodynamic condition as well as the medium composition (pH, substrate concentration)

This study urges the consideration of control of electroactive biofilms. Effective mechanisms to adjust the thickness of the electroactive biofilm should be further investigated in lab‐scale systems with small electrodes to allow for a precise investigation of the impact of the control mechanisms on the electroactive biofilm thickness and its current production. Well‐adjusted protocols need be developed for the periodic application or condition‐based triggering of control mechanisms for a long‐term stable current output. The choice of mechanisms though should not neglect the target of upscaled BES, wherein constructive, operational and economic constraints may limit the sustainable applicability of some mechanisms.

## Author Contributions

**Andreas Netsch:** conceptualization, formal analysis, investigation, writing – original draft preparation. **Inka Latussek:** conceptualization, formal analysis, investigation, writing – review and editing. **Harald Horn:** supervision, funding acquisition, writing – review and editing. **Michael Wagner:** supervision, funding acquisition, writing – review and editing.

## Conflicts of Interest

The authors declare no conflicts of interest.

## Supporting information

SI BioTech BioEng revised.

Figure SI1.

Figure SI2.

Figure SI3.

Figure SI4.

Figure SI5.

Figure SI6.

Figure SI7.

Figure SI8.

Table SI 1.

## Data Availability

The data that support the findings of this study are available from the corresponding author upon reasonable request.

## References

[bit29017-bib-0001] Babanova, S. , J. Jones , S. Phadke , et al. 2020. “Continuous Flow, Large‐Scale, Microbial Fuel Cell System for the Sustained Treatment of Swine Waste.” Water Environment Research 92, no. 1: 60–72. 10.1002/wer.1183. 31306532

[bit29017-bib-0002] Babauta, J. T. , H. D. Nguyen , T. D. Harrington , R. Renslow , and H. Beyenal . 2012. “pH, Redox Potential and Local Biofilm Potential Microenvironments Within *Geobacter Sulfurreducens* Biofilms and Their Roles in Electron Transfer.” Biotechnology and Bioengineering 109, no. 10: 2651–2662. 10.1002/bit.24538. 22549331 PMC3551578

[bit29017-bib-0003] Blatter, M. , L. Delabays , C. Furrer , G. Huguenin , C. P. Cachelin , and F. Fischer . 2021. “Stretched 1000‐L Microbial Fuel Cell.” Journal of Power Sources 483: 229130. 10.1016/j.jpowsour.2020.229130.

[bit29017-bib-0004] Cheng, S. , and B. E. Logan . 2011. “Increasing Power Generation for Scaling Up Single‐Chamber Air Cathode Microbial Fuel Cells.” Bioresource Technology 102, no. 6: 4468–4473. 10.1016/j.biortech.2010.12.104. 21273062

[bit29017-bib-0005] Coppi, M. V. , C. Leang , S. J. Sandler , and D. R. Lovley . 2001. “Development of a Genetic System for *Geobacter Sulfurreducens* .” Applied and Environmental Microbiology 67, no. 7: 3180–3187. 10.1128/AEM.67.7.3180-3187.2001. 11425739 PMC92998

[bit29017-bib-0006] Cussler, E. L. 2009. Diffusion: Mass Transfer in Fluid Systems, 3rd ed. Cambridge University Press.

[bit29017-bib-0007] Dzofou Ngoumelah, D. , T. M. B. Heggeset , T. Haugen , et al. 2024. “Effect of Model Methanogens on the Electrochemical Activity, Stability, and Microbial Community Structure of *Geobacter* spp. Dominated Biofilm Anodes.” NPJ Biofilms and Microbiomes 10, no. 1: 17. 10.1038/s41522-024-00490-z. 38443373 PMC10915144

[bit29017-bib-0008] Fan, L. S. , R. Leyva‐Ramos , K. D. Wisecarver , and B. J. Zehner . 1990. “Diffusion of Phenol Through a Biofilm Grown on Activated Carbon Particles in a Draft‐Tube Three‐Phase Fluidized‐Bed Bioreactor.” Biotechnology and Bioengineering 35, no. 3: 279–286. 10.1002/bit.260350309. 18592520

[bit29017-bib-0009] Franks, A. E. , K. P. Nevin , H. Jia , M. Izallalen , T. L. Woodard , and D. R. Lovley . 2009. “Novel Strategy for Three‐Dimensional Real‐Time Imaging of Microbial Fuel Cell Communities: Monitoring the Inhibitory Effects of Proton Accumulation Within the Anode Biofilm.” Energy & Environmental Science 2, no. 1: 113–119. 10.1039/B816445B.

[bit29017-bib-0010] Georg, S. , I. De Eguren Cordoba , T. Sleutels , P. Kuntke , A. Heijne , and C. J. N. Buisman . 2020. “Competition of Electrogens With Methanogens for Hydrogen in Bioanodes.” Water Research 170: 115292. 10.1016/j.watres.2019.115292. 31778968

[bit29017-bib-0011] Godain, A. , T. M. Vogel , P. Fongarland , and N. Haddour . 2024. “Influence of Shear Stress on Electroactive Biofilm Characteristics and Performance in Microbial Fuel Cells.” Biosensors and Bioelectronics 244: 115806. 10.1016/j.bios.2023.115806. 37944355

[bit29017-bib-0012] Greenman, J. , K. Hewett , and S. Saad . 2020. “Discovery, Development and Exploitation of Steady‐State Biofilms.” Journal of Breath Research 14, no. 4: 044001. 10.1088/1752-7163/abb765. 33021218

[bit29017-bib-0013] Hackbarth, M. , J. Gescher , H. Horn , and J. E. Reiner . 2023. “A Scalable, Rotating Disc Bioelectrochemical Reactor (RDBER) Suitable for the Cultivation of Both Cathodic and Anodic Biofilms.” Bioresource Technology Reports 21: 101357. 10.1016/j.biteb.2023.101357.

[bit29017-bib-0014] Hackbarth, M. , T. Jung , J. E. Reiner , et al. 2020. “Monitoring and Quantification of Bioelectrochemical *Kyrpidia spormannii* Biofilm Development in a Novel Flow Cell Setup.” Chemical Engineering Journal 390: 124604. 10.1016/j.cej.2020.124604.

[bit29017-bib-0015] Haupt, D. R. , L. Landwehr , R. Schumann , et al. 2022. “A New Reactor Concept for Single‐Chamber Microbial Fuel Cells and Possible Anti‐Fouling Strategies for Long‐Term Operation.” Microorganisms 10, no. 12: 2421. 10.3390/microorganisms10122421. 36557674 PMC9784785

[bit29017-bib-0016] Hwang, J. H. , N. Cicek , and J. A. Oleszkiewicz . 2010. “Achieving Biofilm Control in a Membrane Biofilm Reactor Removing Total Nitrogen.” Water Research 44, no. 7: 2283–2291. 10.1016/j.watres.2009.12.022. 20045168

[bit29017-bib-0017] Islam, M. A. , B. Ehiraj , C. K. Cheng , B. N. Dubey , and M. M. R. Khan . 2019. “Biofilm Re‐Vitalization Using Hydrodynamic Shear Stress for Stable Power Generation in Microbial Fuel Cell.” Journal of Electroanalytical Chemistry 844: 14–22. 10.1016/j.jelechem.2019.05.013.

[bit29017-bib-0018] Islam, M. A. , C. W. Woon , B. Ethiraj , C. K. Cheng , A. Yousuf , and M. R. Khan, Md. 2017. “Ultrasound Driven Biofilm Removal for Stable Power Generation in Microbial Fuel Cell.” Energy & Fuels 31, no. 1: 968–976. 10.1021/acs.energyfuels.6b02294.

[bit29017-bib-0019] Jadhav, D. A. , A. D. Chendake , A. Schievano , and D. Pant . 2019. “Suppressing Methanogens and Enriching Electrogens in Bioelectrochemical Systems.” Bioresource Technology 277: 148–156. 10.1016/j.biortech.2018.12.098. 30635224

[bit29017-bib-0020] Jain, A. , G. Gazzola , A. Panzera , M. Zanoni , and E. Marsili . 2011. “Visible Spectroelectrochemical Characterization of Geobacter Sulfurreducens Biofilms on Optically Transparent Indium Tin Oxide Electrode.” Electrochimica Acta 56, no. 28: 10776–10785. 10.1016/j.electacta.2011.02.073.

[bit29017-bib-0021] Kato Marcus, A. , C. I. Torres , and B. E. Rittmann . 2007. “Conduction‐Based Modeling of the Biofilm Anode of a Microbial Fuel Cell.” Biotechnology and Bioengineering 98, no. 6: 1171–1182. 10.1002/bit.21533. 17570714

[bit29017-bib-0022] Ledezma, P. , J. Greenman , and I. Ieropoulos . 2012. “Maximising Electricity Production by Controlling the Biofilm Specific Growth Rate In Microbial Fuel Cells.” Bioresource Technology 118: 615–618. 10.1016/j.biortech.2012.05.054. 22704187

[bit29017-bib-0023] Li, J. , L. Hu , L. Zhang , D. Ye , X. Zhu , and Q. Liao . 2017. “Uneven Biofilm and Current Distribution In Three‐Dimensional Macroporous Anodes of Bio‐Electrochemical Systems Composed of Graphite Electrode Arrays.” Bioresource Technology 228: 25–30. 10.1016/j.biortech.2016.12.092. 28056366

[bit29017-bib-0024] Malvankar, N. S. , J. Lau , K. P. Nevin , A. E. Franks , M. T. Tuominen , and D. R. Lovley . 2012. “Electrical Conductivity in a Mixed‐Species Biofilm.” Applied and Environmental Microbiology 78, no. 16: 5967–5971. 10.1128/AEM.01803-12. 22706052 PMC3406156

[bit29017-bib-0025] Malvankar, N. S. , M. Vargas , K. P. Nevin , et al. 2011. “Tunable Metallic‐Like Conductivity in Microbial Nanowire Networks.” Nature Nanotechnology 6, no. 9: 573–579. 10.1038/nnano.2011.119. 21822253

[bit29017-bib-0026] Molenaar, S. D. , T. Sleutels , J. Pereira , et al. 2018. “In Situ Biofilm Quantification in Bioelectrochemical Systems by Using Optical Coherence Tomography.” Chemsuschem 11, no. 13: 2171–2178. 10.1002/cssc.201800589. 29693330 PMC6055872

[bit29017-bib-0027] Netsch, A. , H. Horn , and M. Wagner . 2021. “On‐Line Monitoring of Biofilm Accumulation on Graphite‐Polypropylene Electrode Material Using a Heat Transfer Sensor.” Biosensors 12, no. 1: 18. 10.3390/bios12010018. 35049646 PMC8773567

[bit29017-bib-0028] Netsch, A. , S. Sen , H. Horn , and M. Wagner . 2025. “In Situ Biofilm Monitoring Using a Heat Transfer Sensor: The Impact of Flow Velocity in a Pipe and Planar System.” Biosensors 15, no. 2: 93. 10.3390/bios15020093. 39996995 PMC11853227

[bit29017-bib-0029] Oliveira, V. B. , M. Simões , L. F. Melo , and A. M. F. R. Pinto . 2013. “A 1D Mathematical Model for a Microbial Fuel Cell.” Energy 61: 463–471. 10.1016/j.energy.2013.08.055.

[bit29017-bib-0030] Patil, S. A. , F. Harnisch , C. Koch , et al. 2011. “Electroactive Mixed Culture Derived Biofilms in Microbial Bioelectrochemical Systems: The Role of pH on Biofilm Formation, Performance and Composition.” Bioresource Technology 102, no. 20: 9683–9690. 10.1016/j.biortech.2011.07.087. 21855323

[bit29017-bib-0031] Pereira, J. , S. Pang , C. Borsje , T. Sleutels , B. Hamelers , and A. Ter Heijne . 2022. “Real‐Time Monitoring of Biofilm Thickness Allows for Determination of Acetate Limitations in Bio‐Anodes.” Bioresource Technology Reports 18: 101028. 10.1016/j.biteb.2022.101028.

[bit29017-bib-0032] Pereira, J. , G. Wang , T. Sleutels , B. Hamelers , and A. Ter Heijne . 2022. “Maximum Thickness of Non‐Buffer Limited Electro‐Active Biofilms Decreases at Higher Anode Potentials.” Biofilm 4: 100092. 10.1016/j.bioflm.2022.100092. 36425753 PMC9678801

[bit29017-bib-0033] Philipp, L.‐A. , K. Bühler , R. Ulber , and J. Gescher . 2024. “Beneficial Applications of Biofilms.” Nature Reviews Microbiology 22, no. 5: 276–290. 10.1038/s41579-023-00985-0. 37957398

[bit29017-bib-0034] Picioreanu, C. , M. C. M. Van Loosdrecht , T. P. Curtis , and K. Scott . 2010. “Model Based Evaluation of the Effect of pH and Electrode Geometry on Microbial Fuel Cell Performance.” Bioelectrochemistry 78, no. 1: 8–24. 10.1016/j.bioelechem.2009.04.009. 19523880

[bit29017-bib-0035] Pinck, S. , L. M. Ostormujof , S. Teychené , and B. Erable . 2020. “Microfluidic Microbial Bioelectrochemical Systems: An Integrated Investigation Platform for a More Fundamental Understanding of Electroactive Bacterial Biofilms.” Microorganisms 8, no. 11: 1841. 10.3390/microorganisms8111841. 33238493 PMC7700166

[bit29017-bib-0036] Read, S. T. , P. Dutta , P. L. Bond , J. Keller , and K. Rabaey . 2010. “Initial Development and Structure of Biofilms on Microbial Fuel Cell Anodes.” BMC Microbiology 10, no. 1: 98. 10.1186/1471-2180-10-98. 20356407 PMC2858741

[bit29017-bib-0037] Reguera, G. , K. P. Nevin , J. S. Nicoll , S. F. Covalla , T. L. Woodard , and D. R. Lovley . 2006. “Biofilm and Nanowire Production Leads to Increased Current in *Geobacter Sulfurreducens* Fuel Cells.” Applied and Environmental Microbiology 72, no. 11: 7345–7348. 10.1128/AEM.01444-06. 16936064 PMC1636155

[bit29017-bib-0038] Renslow, R. , J. Babauta , A. Dohnalkova , et al. 2013. “Metabolic Spatial Variability in Electrode‐Respiring *Geobacter Sulfurreducens* Biofilms.” Energy & Environmental Science 6, no. 6: 1827–1836. 10.1039/c3ee40203g. 23930138 PMC3733395

[bit29017-bib-0039] Renslow, R. S. , P. D. Majors , J. S. McLean , J. K. Fredrickson , B. Ahmed , and H. Beyenal . 2010. “In Situ Effective Diffusion Coefficient Profilesin Live Biofilms Using Pulsed‐Field Gradient Nuclear Magnetic Resonance.” Biotechnology and Bioengineering 106, no. 6: 928–937. 10.1002/bit.22755. 20589671 PMC2898744

[bit29017-bib-0040] Santoro, C. , S. Babanova , P. Cristiani , et al. 2021. “Front Cover: How Comparable Are Microbial Electrochemical Systems Around the Globe? An Electrochemical and Microbiological Cross‐Laboratory Study (Chemsuschem 11/2021).” Chemsuschem 14, no. 11: 2262. 10.1002/cssc.202100825. 34002490

[bit29017-bib-0041] Schröder, U. 2007. “Anodic Electron Transfer Mechanisms in Microbial Fuel Cells and Their Energy Efficiency.” Physical Chemistry Chemical Physics: PCCP 9, no. 21: 2619–2629. 10.1039/B703627M. 17627307

[bit29017-bib-0042] Sleutels, T. , S. Molenaar , A. Heijne , and C. Buisman . 2016. “Low Substrate Loading Limits Methanogenesis and Leads to High Coulombic Efficiency in Bioelectrochemical Systems.” Microorganisms 4, no. 1: 7. 10.3390/microorganisms4010007. 27681899 PMC5029512

[bit29017-bib-0043] Sun, D. , J. Chen , H. Huang , W. Liu , Y. Ye , and S. Cheng . 2016. “The Effect of Biofilm Thickness on Electrochemical Activity of *Geobacter Sulfurreducens* .” International Journal of Hydrogen Energy 41, no. 37: 16523–16528. 10.1016/j.ijhydene.2016.04.163.

[bit29017-bib-0044] Ullah, Z. , and S. Zeshan . 2020. “Effect of Substrate Type and Concentration on the Performance of a Double Chamber Microbial Fuel Cell.” Water Science and Technology 81, no. 7: 1336–1344. 10.2166/wst.2019.387. 32616686

[bit29017-bib-0045] Wagner, M. , and H. Horn . 2017. “Optical Coherence Tomography in Biofilm Research: A Comprehensive Review.” Biotechnology and Bioengineering 114, no. 7: 1386–1402. 10.1002/bit.26283. 28266013

[bit29017-bib-0046] Wei, C.‐H. , X.‐Y. Zhai , Y.‐D. Jiang , et al. 2024. “Simultaneous Carbon, Nitrogen and Phosphorus Removal in Sequencing Batch Membrane Aerated Biofilm Reactor With Biofilm Thickness Control via Air Scouring Aided by Computational Fluid Dynamics.” Bioresource Technology 409: 131267. 10.1016/j.biortech.2024.131267. 39142417

[bit29017-bib-0047] Wu, Y. , F. Li , T. Liu , R. Han , and X. Luo . 2016. “pH Dependence of Quinone‐Mediated Extracellular Electron Transfer in a Bioelectrochemical System.” Electrochimica Acta 213: 408–415. 10.1016/j.electacta.2016.07.122.

[bit29017-bib-0048] Yan, X. , Q. Du , Q. Mu , et al. 2021. “Long‐Term Succession Shows Interspecies Competition of *Geobacter* in Exoelectrogenic Biofilms.” Environmental Science & Technology 55, no. 21: 14928–14937. 10.1021/acs.est.1c03010. 34676765

[bit29017-bib-0049] Yang, W. , J. Li , Q. Fu , et al. 2021. “Minimizing Mass Transfer Losses in Microbial Fuel Cells: Theories, Progresses and Prospectives.” Renewable and Sustainable Energy Reviews 136: 110460. 10.1016/j.rser.2020.110460.

[bit29017-bib-0050] Zhang, E. , F. Wang , Q. Yu , K. Scott , X. Wang , and G. Diao . 2017. “Durability and Regeneration of Activated Carbon Air‐Cathodes in Long‐Term Operated Microbial Fuel Cells.” Journal of Power Sources 360: 21–27. 10.1016/j.jpowsour.2017.05.119.

