# Supplementary material for: Detecting Excess Biofilm Thickness in Microbial Electrolysis Cells by Real‐Time In‐Situ Biofilm Monitoring
Source: Biotechnol Bioeng. 2025 May 2;122(8):2049–62. doi: 10.1002/bit.29017 (PMC12235218; doi:10.1002/bit.29017)

# Supplementary Information:

# Detecting excess biofilm thickness in microbial electrolysis cells by real-time in-situ biofilm monitoring

**Authors:** Andreas Netsch^1,2^, Inka Latussek^1,2^, Harald Horn^1,2^, Michael Wagner^2,3^

**Affiliations:**
^1^DVGW Research Center, Water Chemistry and Water Technology, Engler-Bunte-Ring 9a, 76131 Karlsruhe, Germany

^2^Engler-Bunte-Institut, Water Chemistry and Water Technology, Karlsruhe Institute of Technology (KIT), Engler-Bunte-Ring 9a, 76131 Karlsruhe, Germany

^3^Institute for Biological Interfaces 1 (IBG-1), Institute for Biological Interfaces (IBG), Karlsruhe Institute of Technology (KIT), Hermann-von-Helmholtz-Platz 1, 76344 Eggenstein-Leopoldshafen, Germany

Development of current density and mean biofilm thickness during the batch phase (Day 0-7)


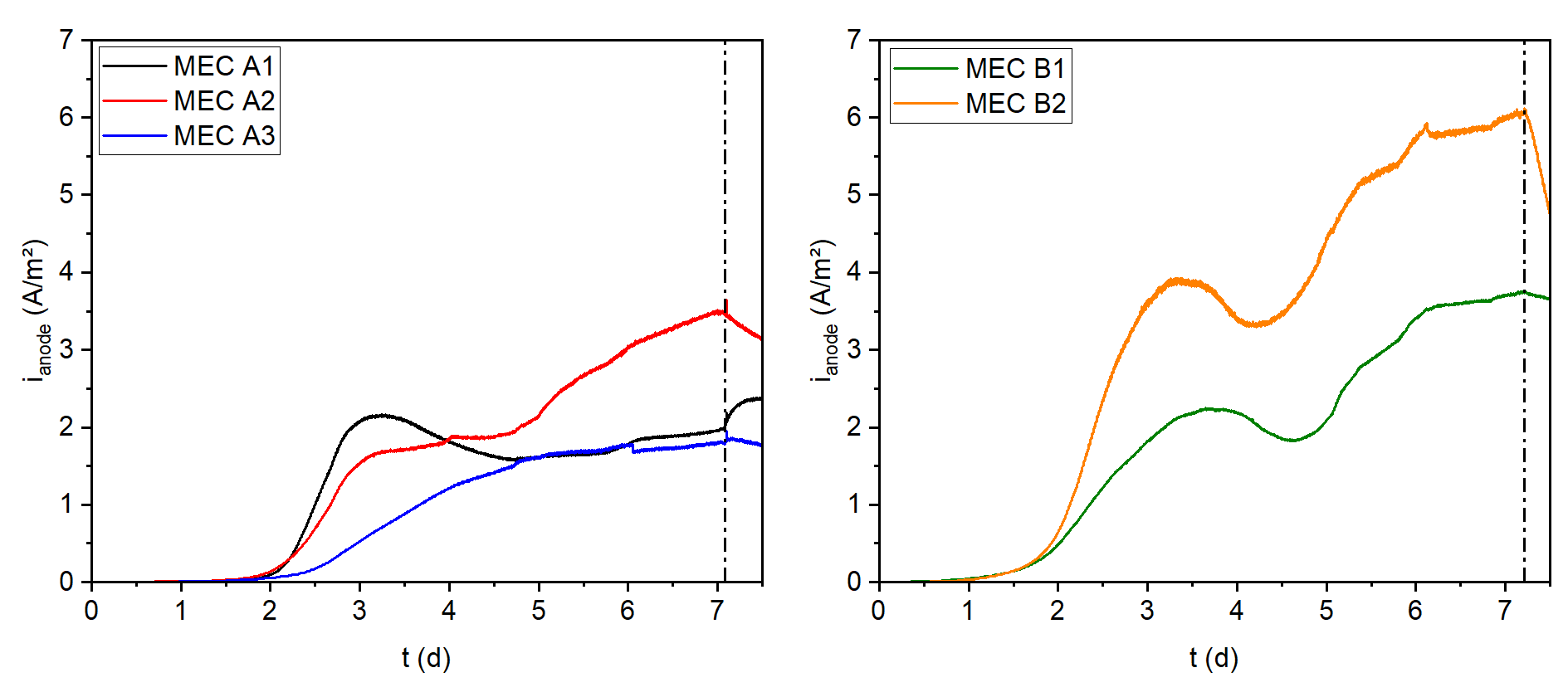


Figure SI-1: Development of the current density in the MECs during the batch period (Day 0-7). The dashed line indicates the start of the continuous operation.


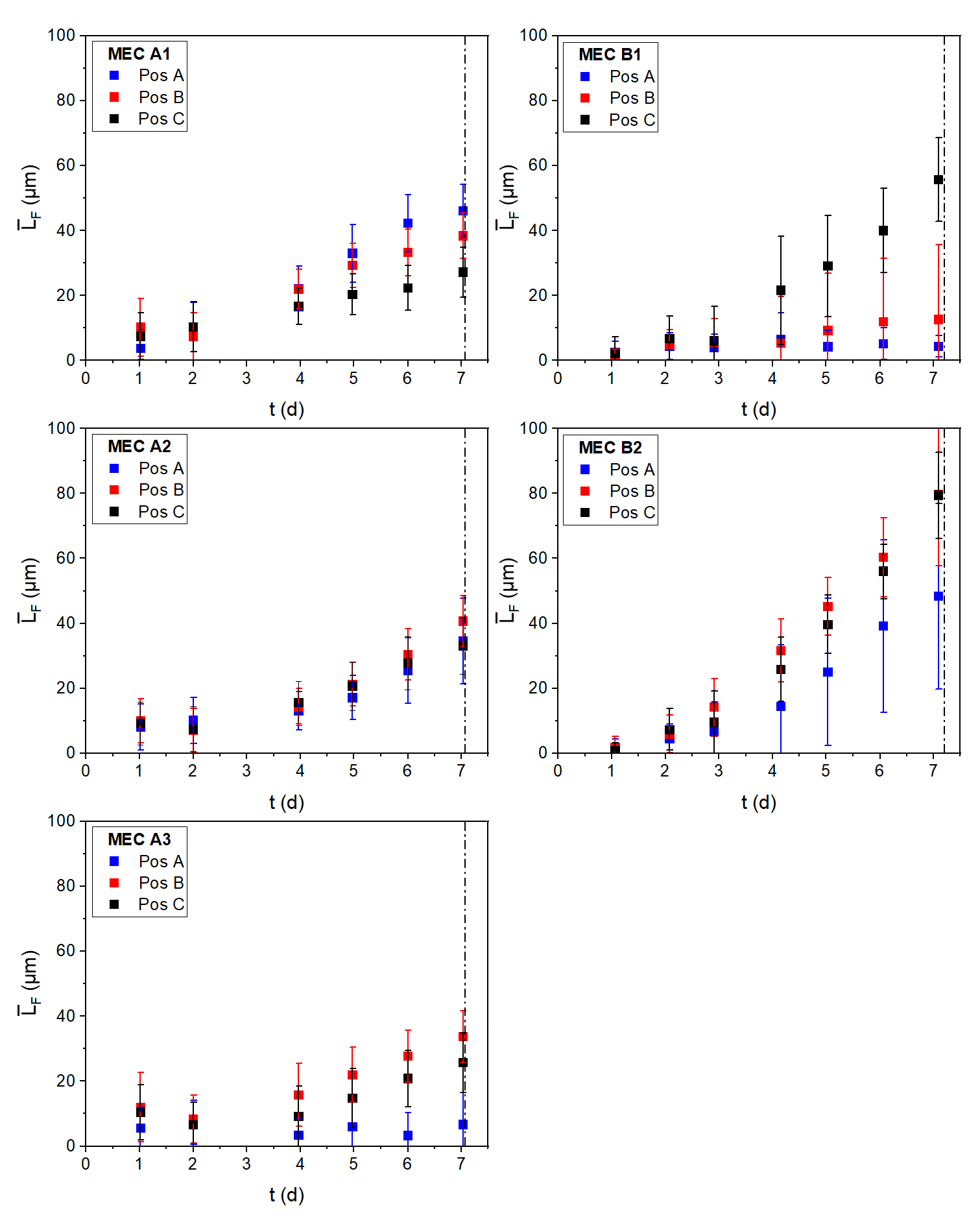


Figure SI-2 shows the mean biofilm thickness separated for the three imaging positions (A-C, compare Figure 1) for each of the MECs. Note, especially in MEC A3 and B1 the lower mean biofilm thickness at the position C in comparison to the position A.


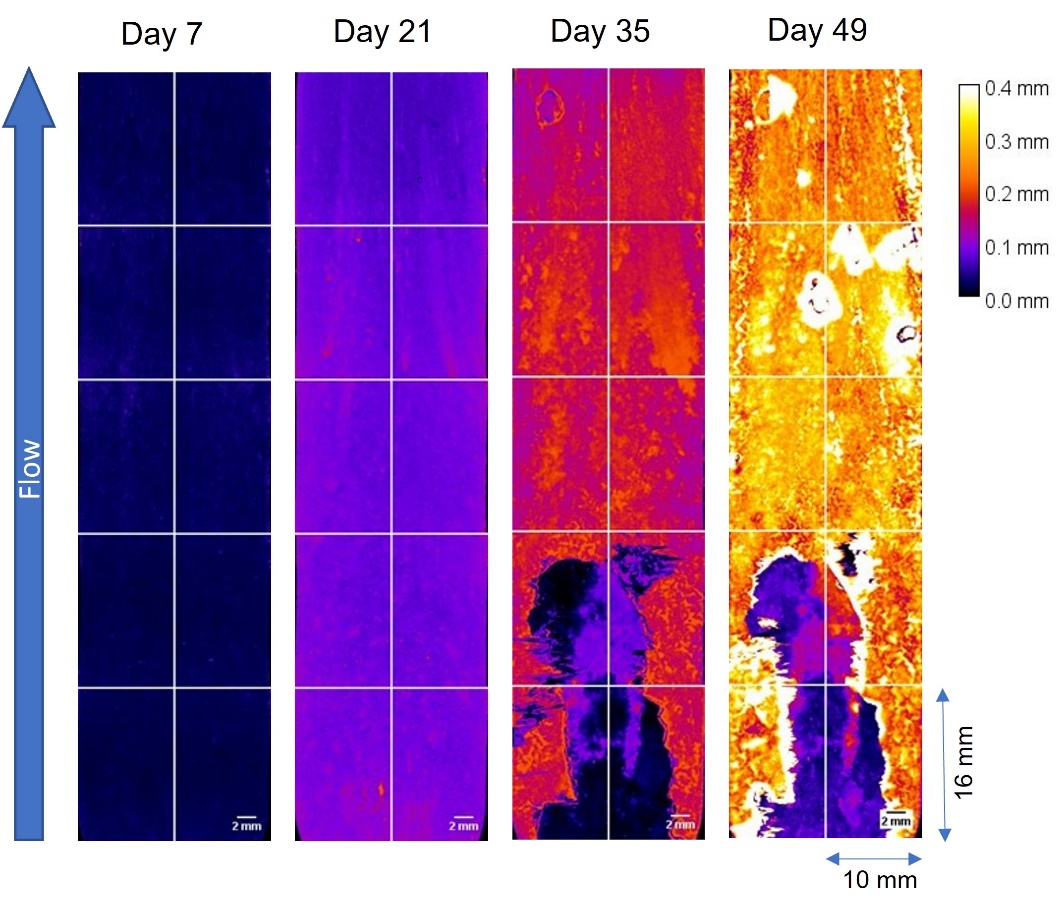

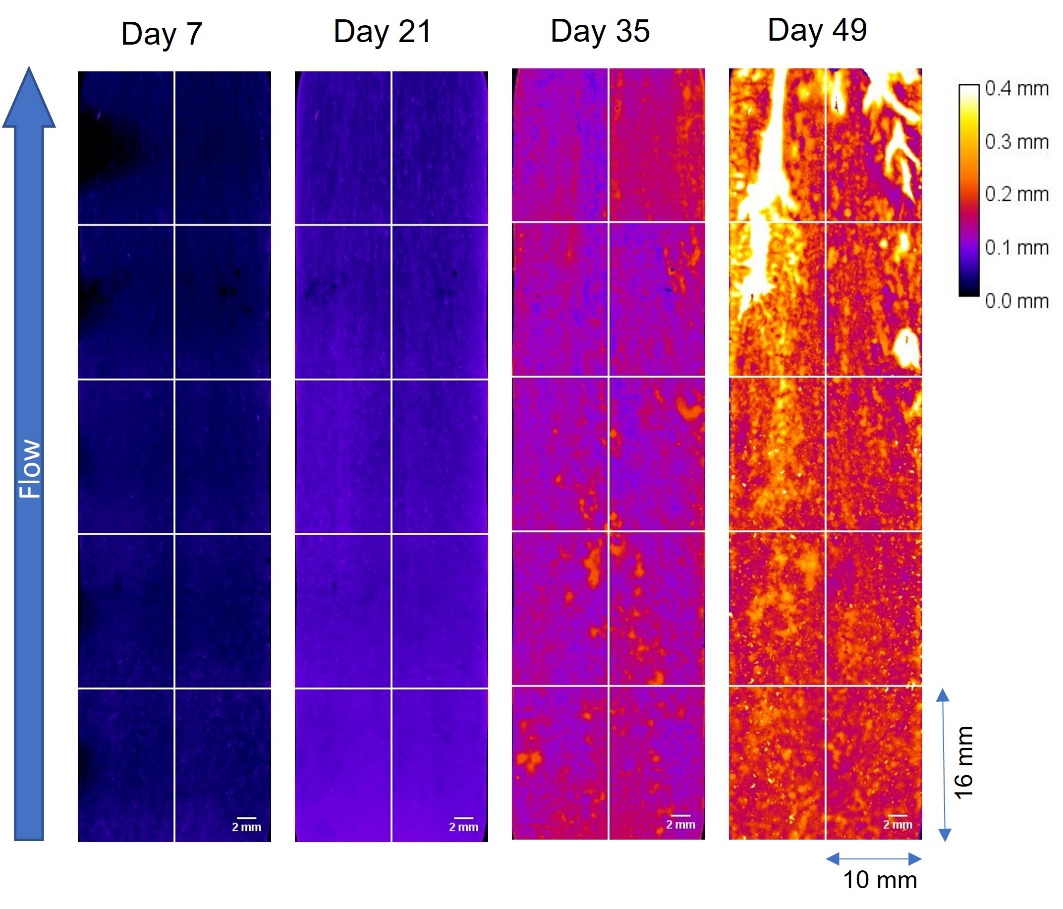
Full scan Height Maps

Figure SI-4: Height maps showing the distance of the bulk-biofilm interface from the electrode substratum of MEC A2 (see Table 1) For Days 7, 21, 35 and 49 a full scan displaying approx. 82 % of the electrode were taken. The height map displays the thickness of the biofilm according to the heat map for the range of 0-400 µm. The direction of flow was from bottom to top

Figure SI-3: Height maps showing the distance of the bulk-biofilm interface from the electrode substratum of MEC A1 (see Table 1) For Days 7, 21, 35 and 49 a full scan displaying approx. 82 % of the electrode were taken. The height map displays the thickness of the biofilm according to the heat map for the range of 0-400 µm. The direction of flow was from bottom to top


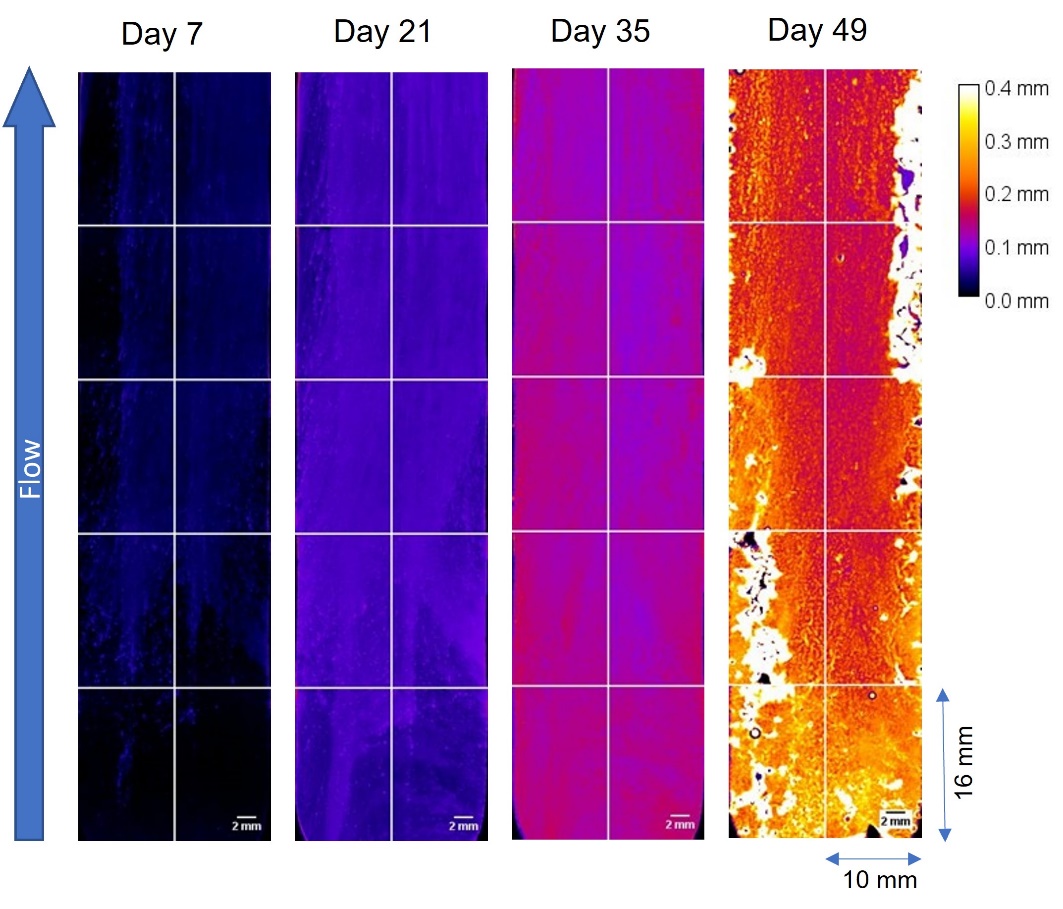

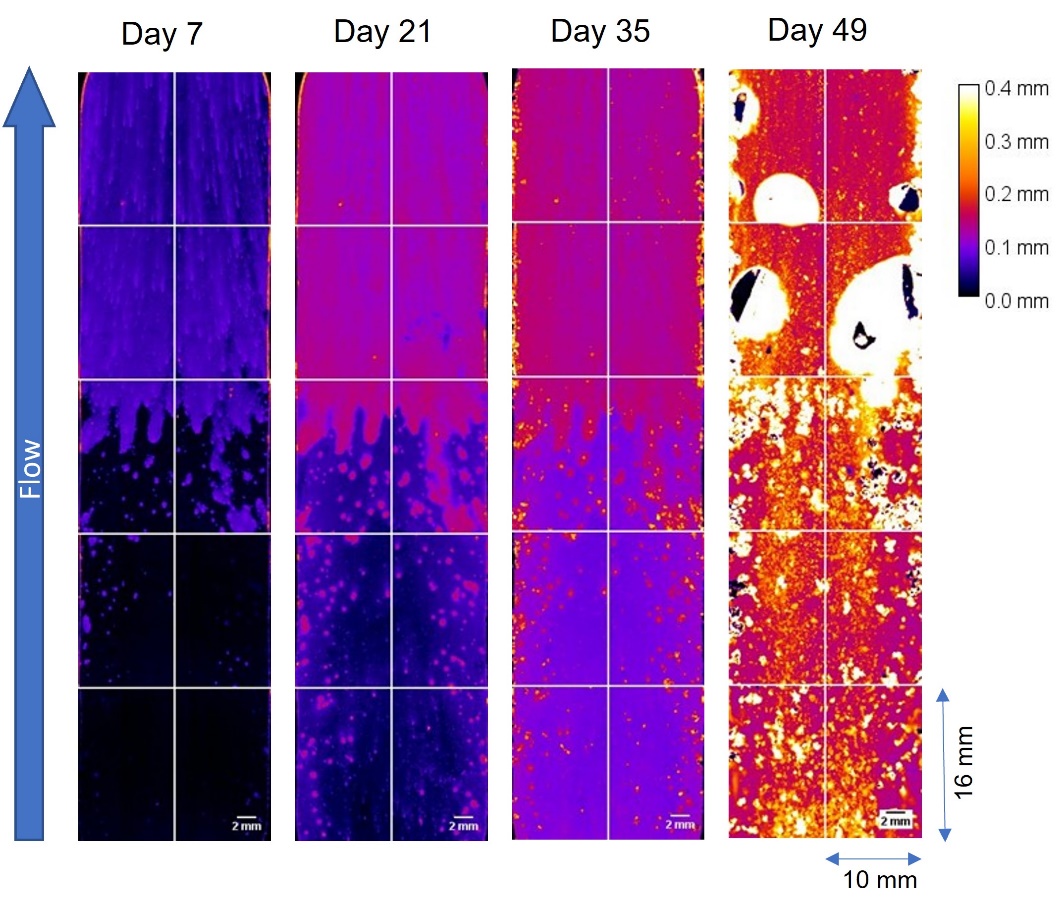


Figure SI-6: Height maps showing the distance of the bulk-biofilm interface from the electrode substratum of MEC B1 (see Table 1) For Days 7, 21, 35 and 49 a full scan displaying approx. 82 % of the electrode were taken. The height map displays the thickness of the biofilm according to the heat map for the range of 0-400 µm. The direction of flow was from bottom to top

Figure SI-5: Height maps showing the distance of the bulk-biofilm interface from the electrode substratum of MEC A3 (see Table 1) For Days 7, 21, 35 and 49 a full scan displaying approx. 82 % of the electrode were taken. The height map displays the thickness of the biofilm according to the heat map for the range of 0-400 µm. The direction of flow was from bottom to top

Specific volumetric current production of the electroactive biofilm:


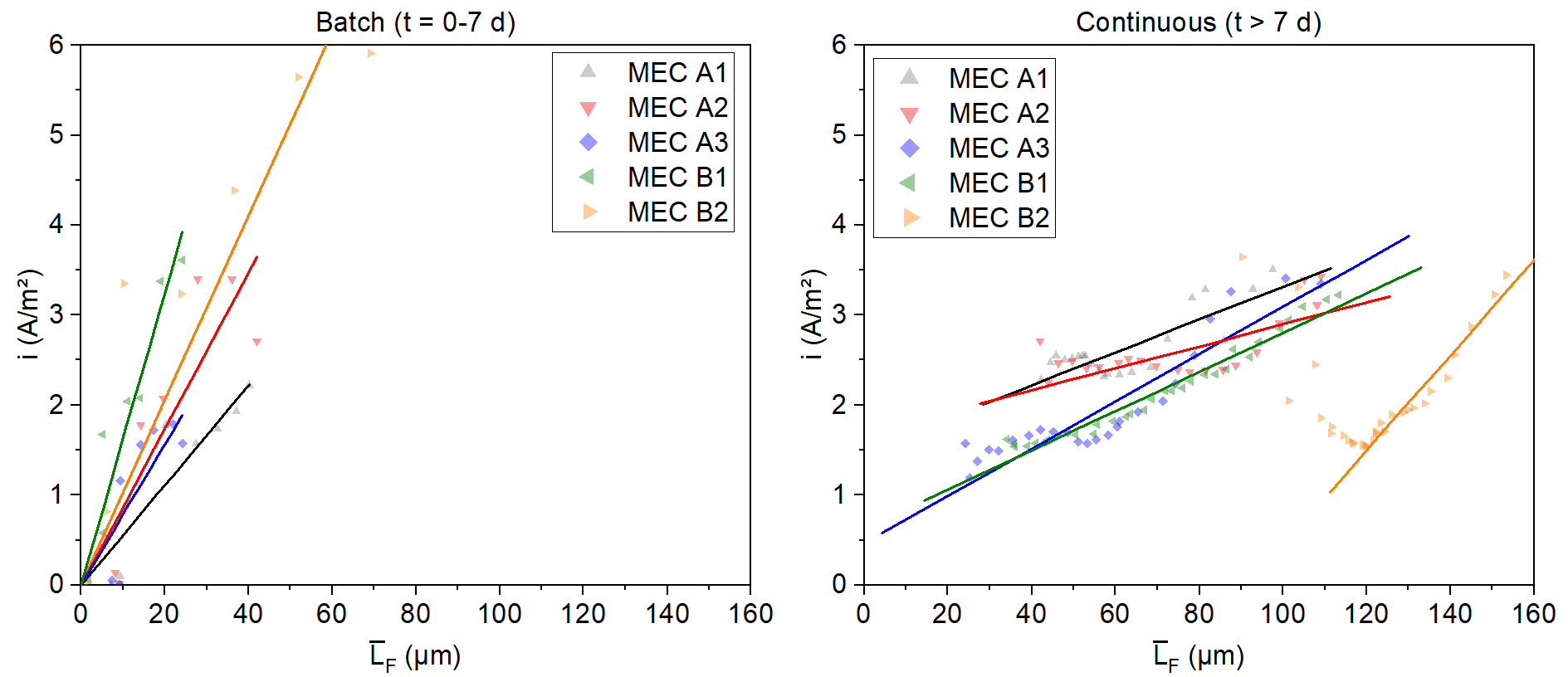


Figure SI-7: Correlation of the current density with the mean biofilm thickness during the growth phase in batch mode (Day 0-7) and during continuous operation until the maximum current production. The lines indicate the slope of the specific current production per volume of biomass.

Table SI-1: Comparison of the specific volumetric current production i_F_ of the electroactive biofilm during the growth in batch mode (Day 0-7) and during the continuous mode for Day 7 to the maximum current production

|  | Batch | | Continuous | |
| --- | --- | --- | --- | --- |
|  | i_F_ (A/mm³) | R² | i_F_ (A/mm³) | R² |
| MEC A1 | 55.456 | 0.95 | 18.23 | 0.67 |
| MEC A2 | 86.881 | 0.91 | 12.18 | 0.59 |
| MEC A3 | 78.113 | 0.88 | 26.22 | 0.86 |
| MEC B1 | 102.712 | 0.93 | 21.8 | 0.96 |
| MEC B2 | 162.217 | 0.97 | 53.02 | 0.96 |
| Mean | 97.076 ± 32.84 |  | 26.29 ± 12.902 |  |

Representativeness of OCT imaging positions:

Figure SI-8: (left) Comparison of the mean biofilm thickness from imaging position 5 with the mean biofilm thickness from full scan images - showing the representativeness of the biofilm sensor measurement (right) Comparison of the mean biofilm thickness from imaging positions 1, 3 and 5 with the mean biofilm thickness from full scan images - showing the representativeness of the daily imaging scheme for the correlation of the current density with the mean biofilm thickness


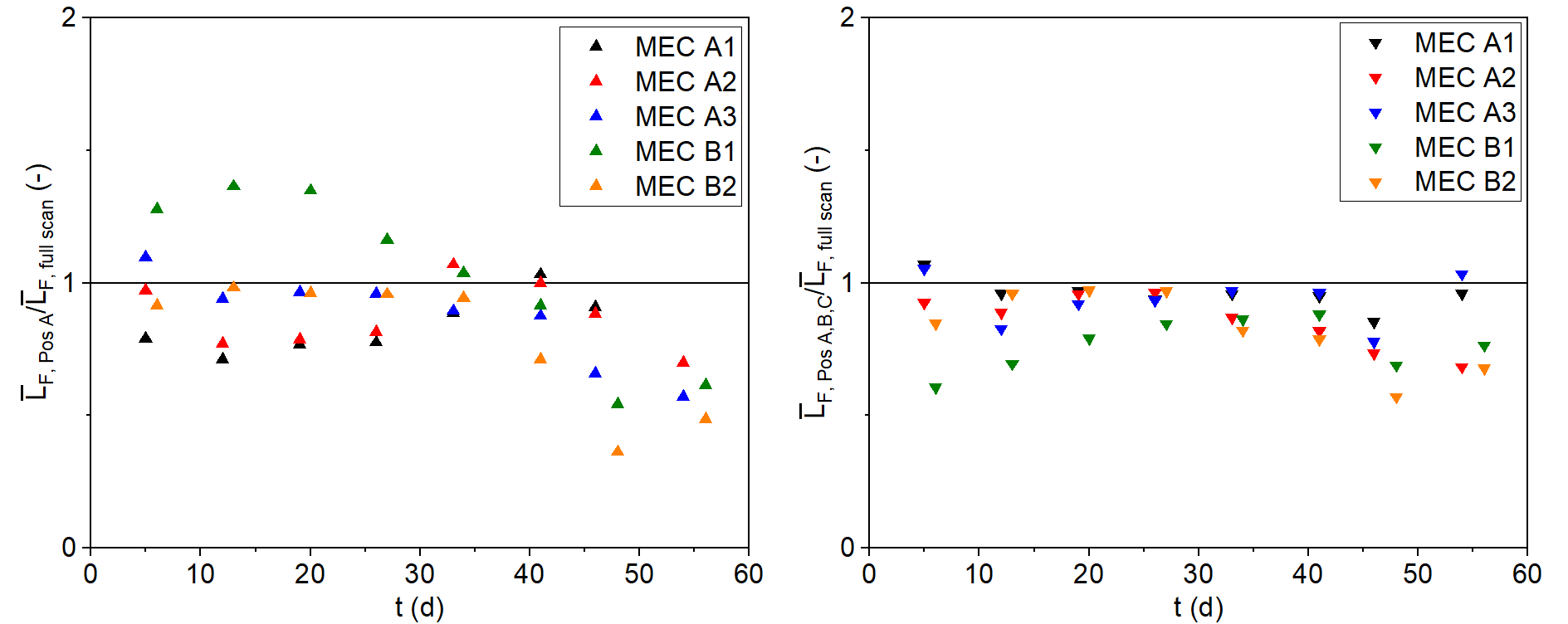

Supplement: Supplementary file 1 — SI BioTech BioEng revised. [file BIT-122-2049-s009.docx]
