# Supplementary figures and images for: Detecting Excess Biofilm Thickness in Microbial Electrolysis Cells by Real‐Time In‐Situ Biofilm Monitoring
Source: Biotechnol Bioeng. 2025 May 2;122(8):2049–62. doi: 10.1002/bit.29017 (PMC12235218; doi:10.1002/bit.29017)

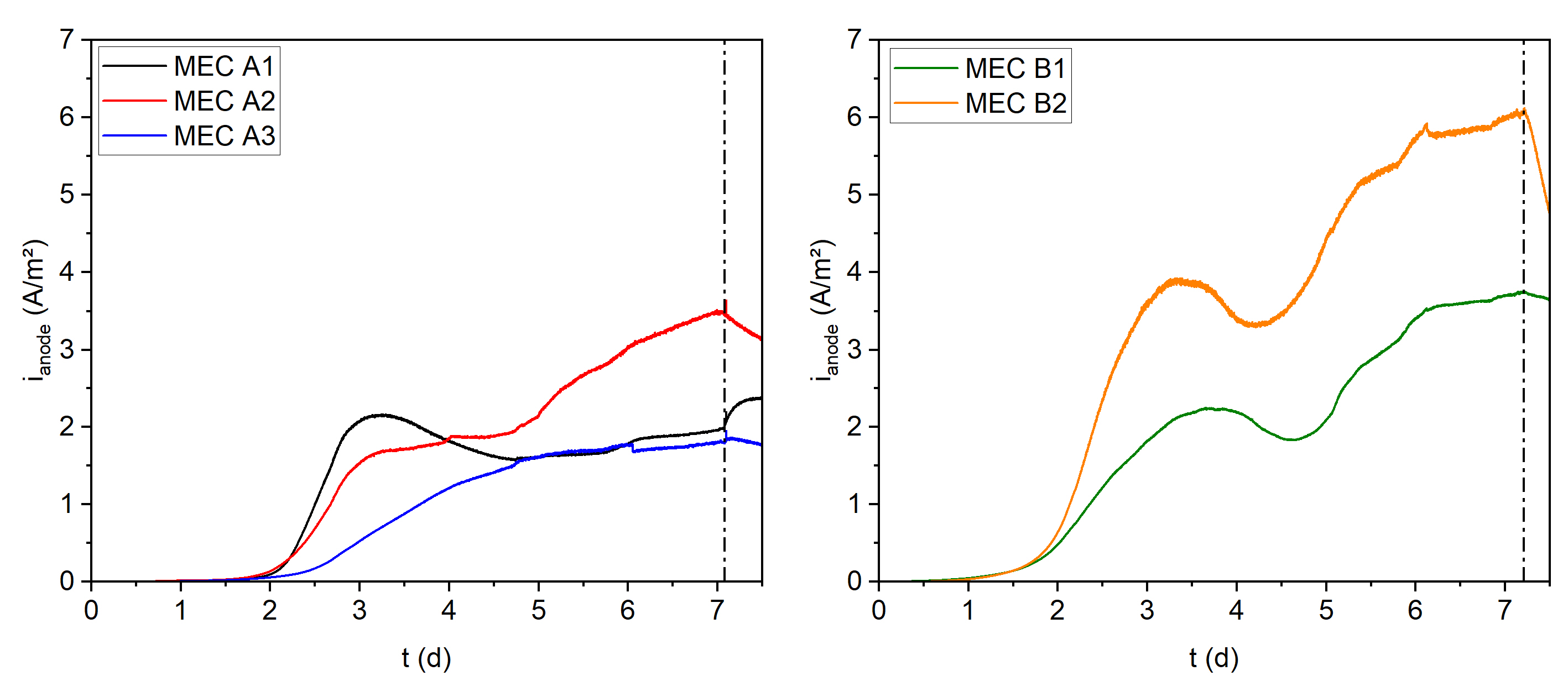

Supplement: Supplementary file 2 — Figure SI1. [file BIT-122-2049-s010.jpg]

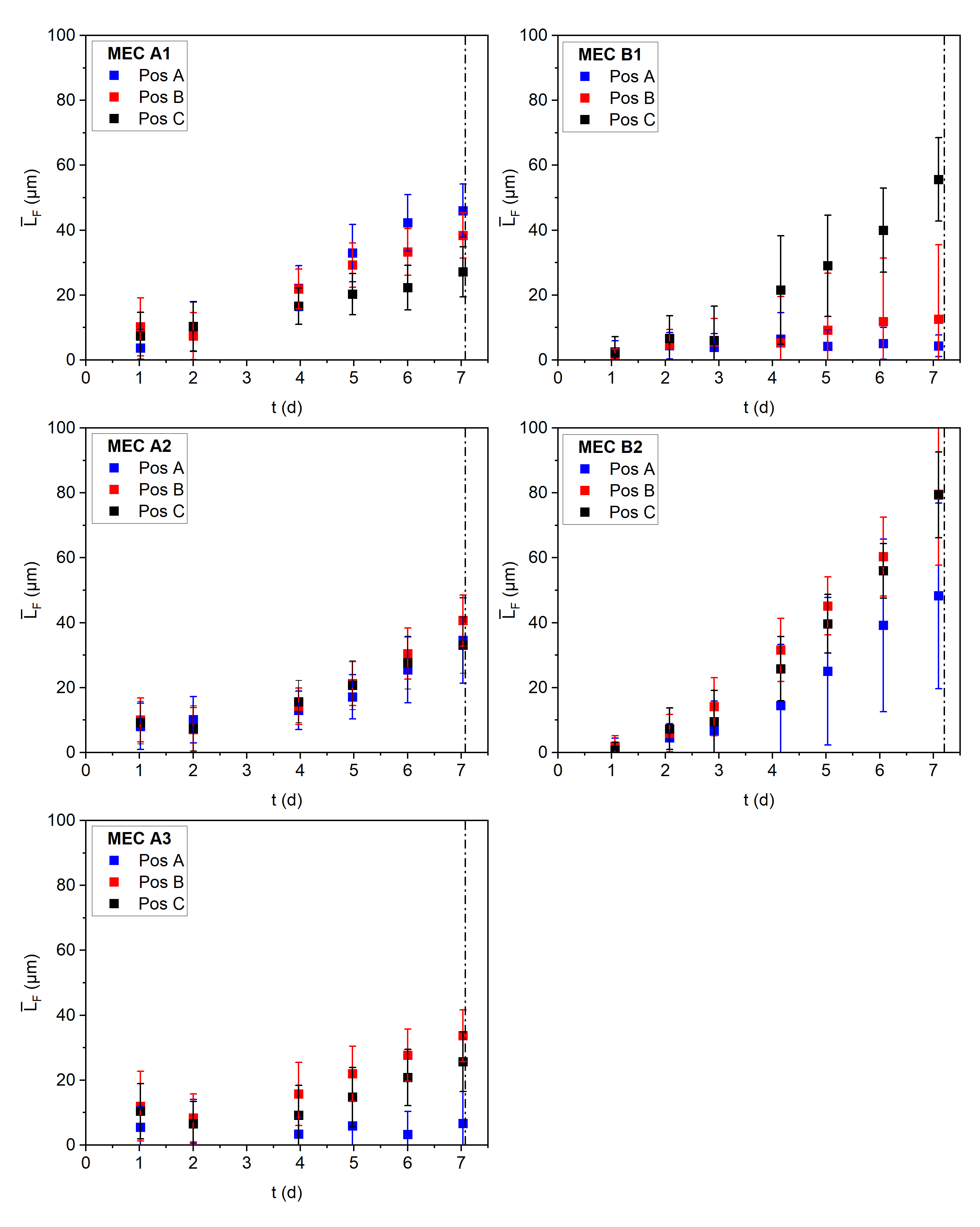

Supplement: Supplementary file 3 — Figure SI2. [file BIT-122-2049-s001.jpg]

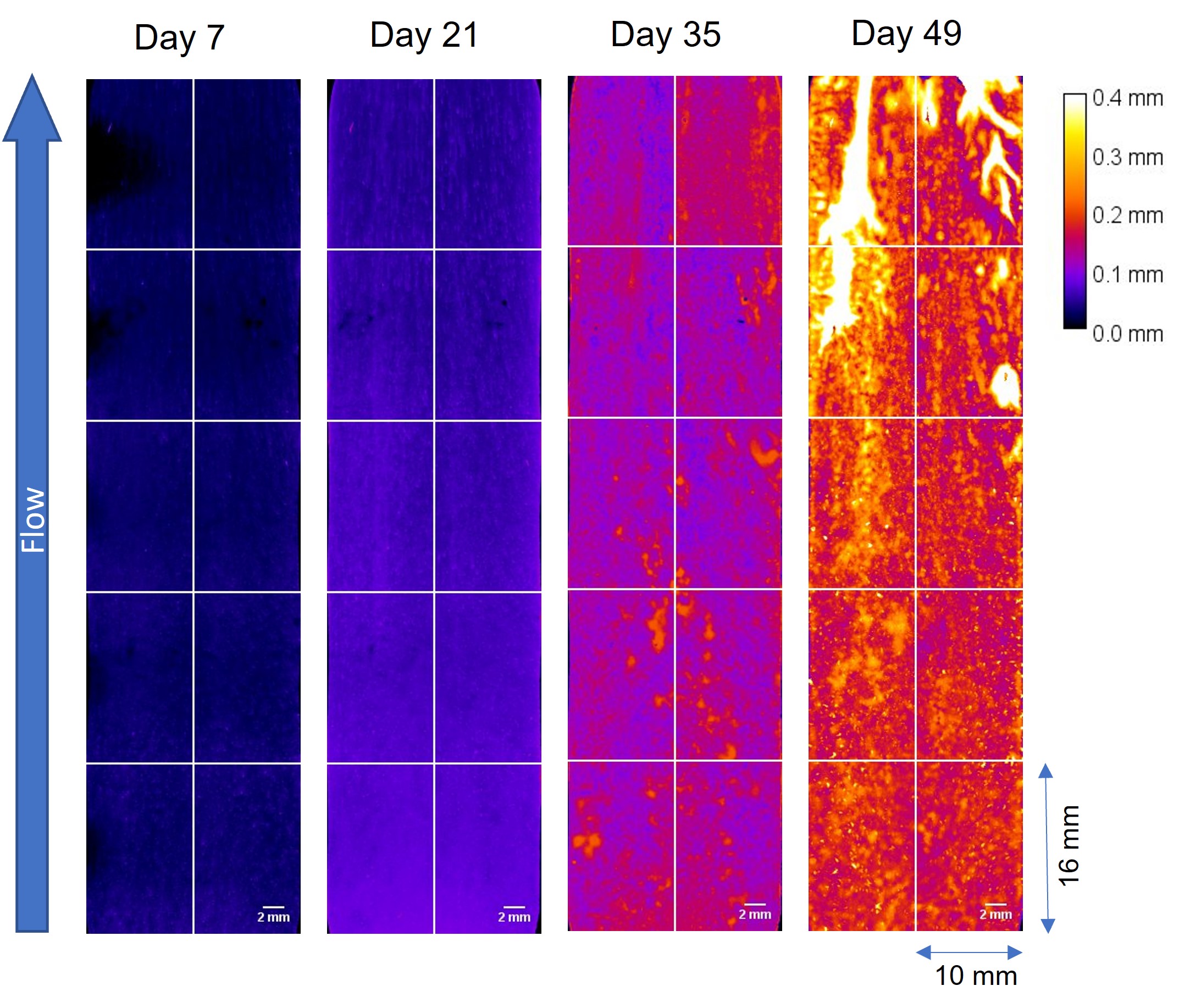

Supplement: Supplementary file 4 — Figure SI3. [file BIT-122-2049-s005.jpg]

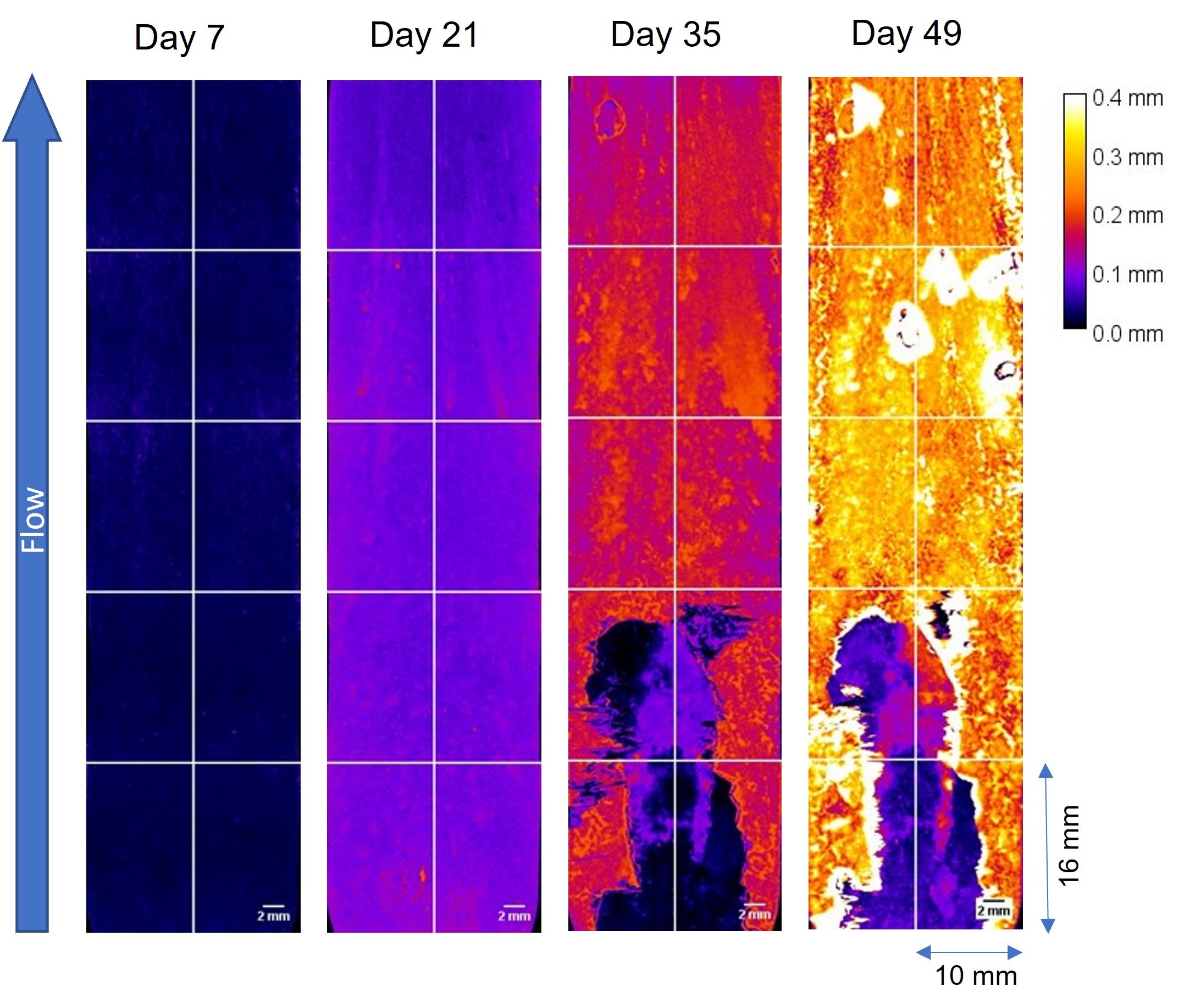

Supplement: Supplementary file 5 — Figure SI4. [file BIT-122-2049-s008.jpg]

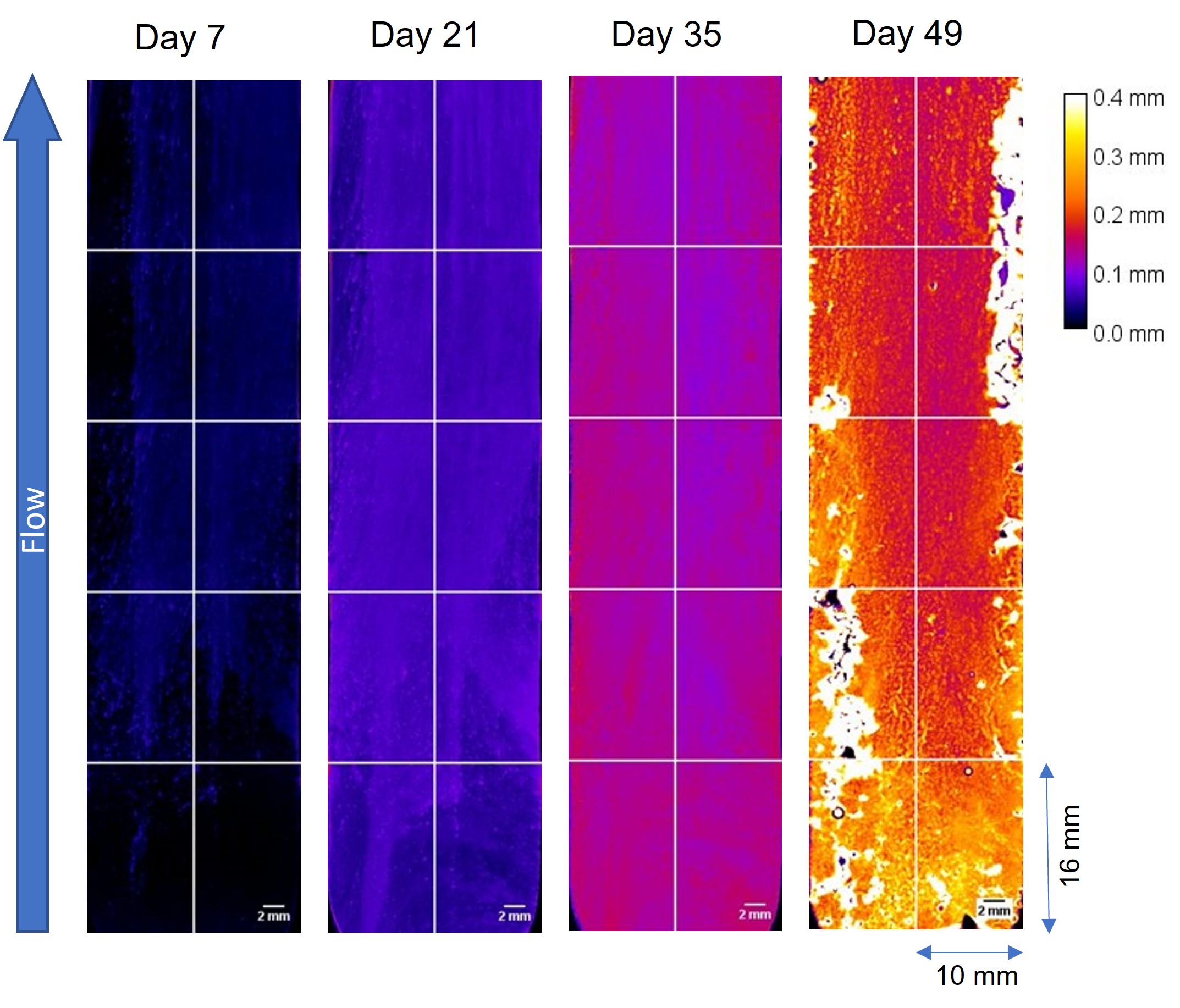

Supplement: Supplementary file 6 — Figure SI5. [file BIT-122-2049-s007.jpg]

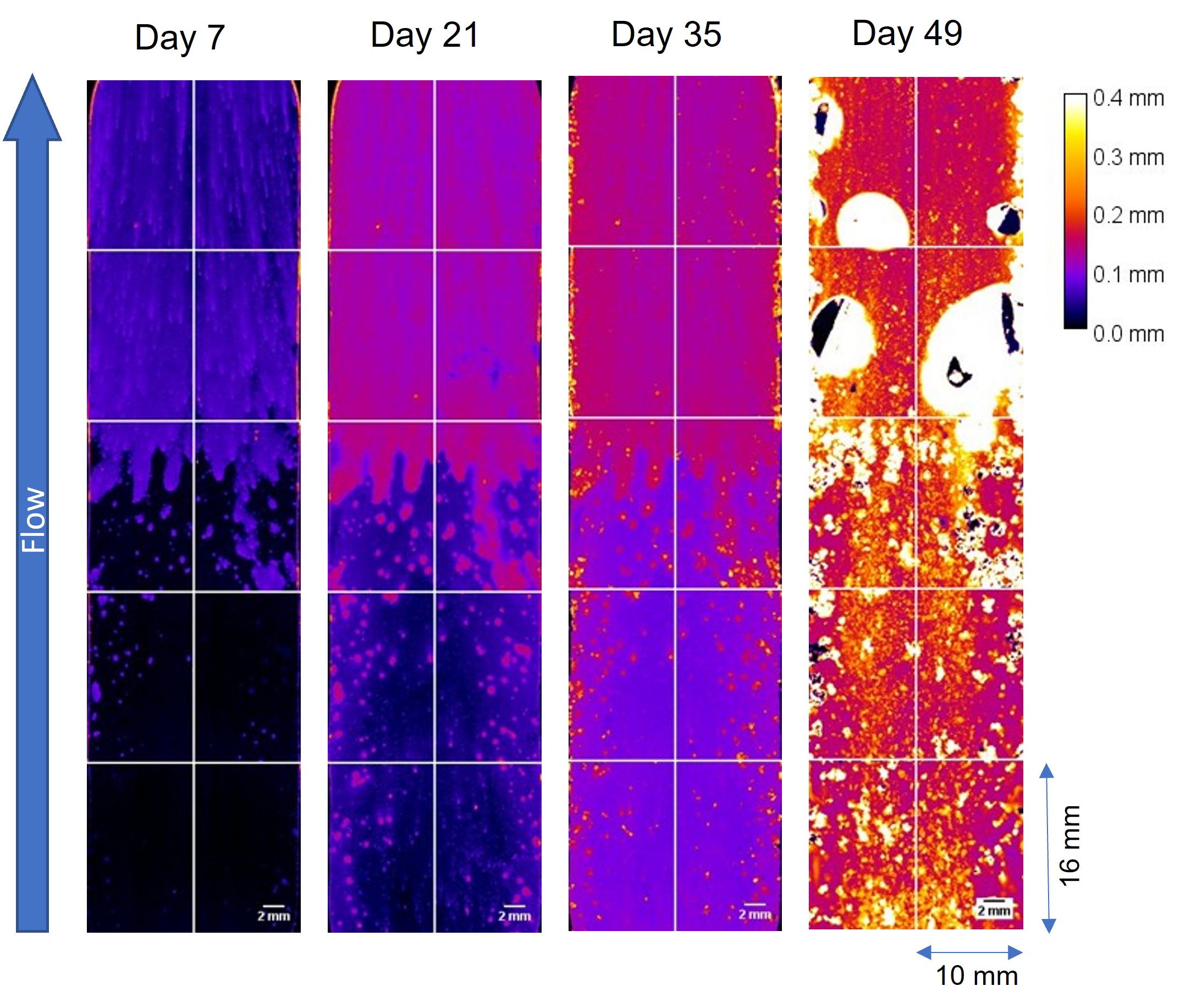

Supplement: Supplementary file 7 — Figure SI6. [file BIT-122-2049-s002.jpg]

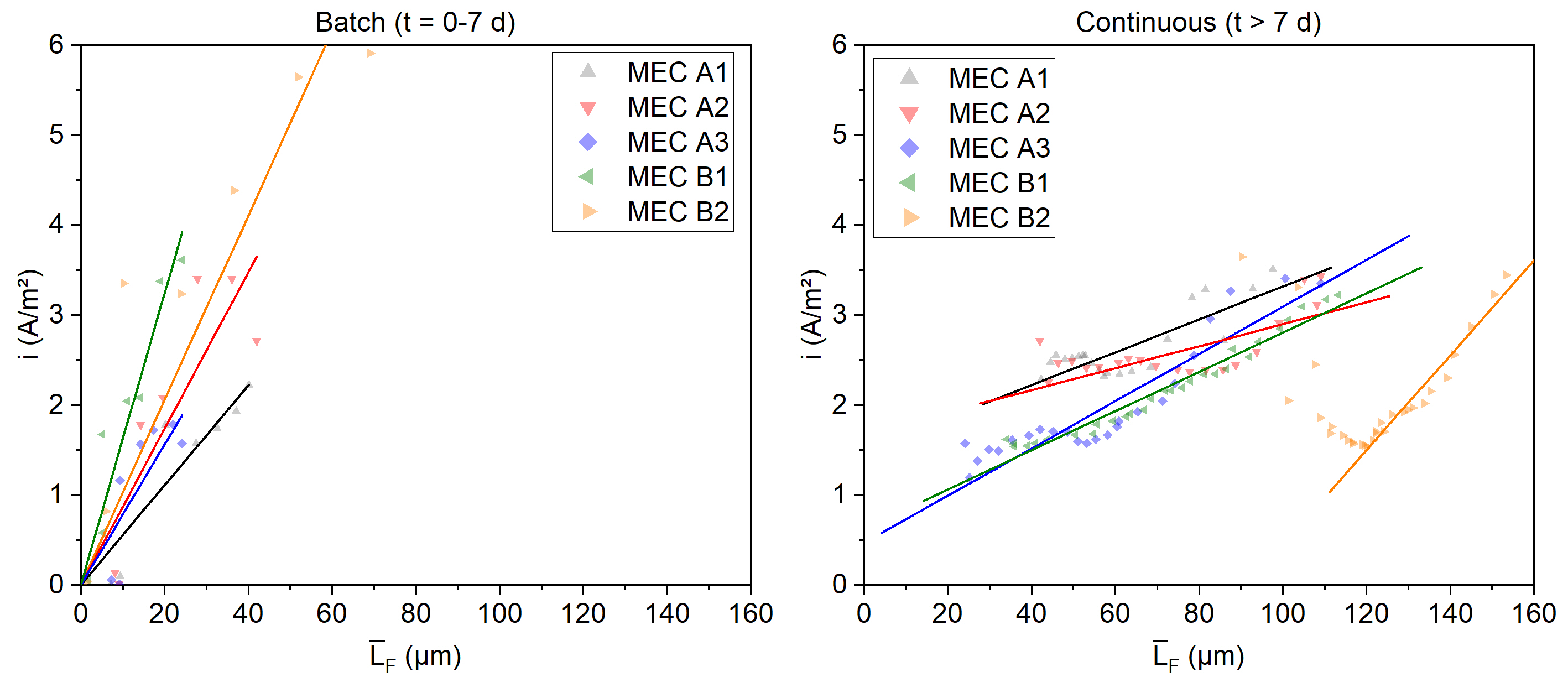

Supplement: Supplementary file 8 — Figure SI7. [file BIT-122-2049-s003.jpg]

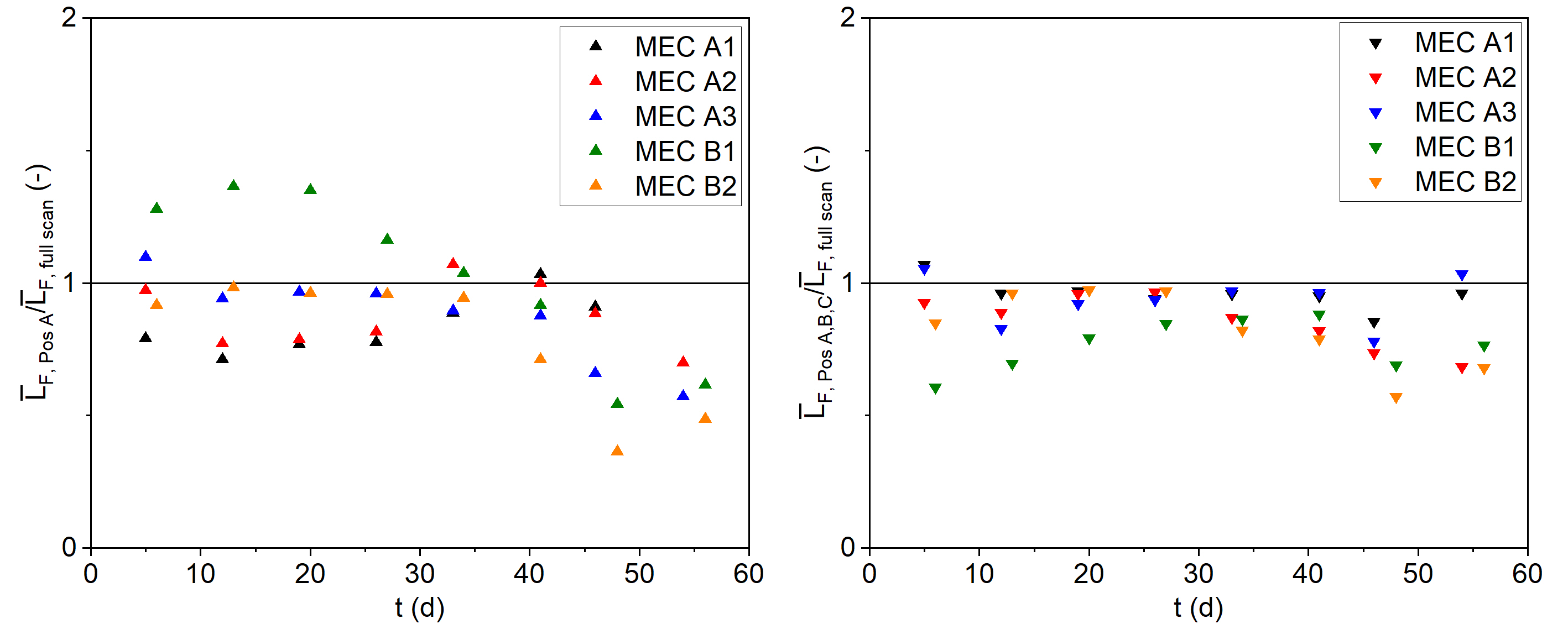

Supplement: Supplementary file 9 — Figure SI8. [file BIT-122-2049-s004.jpg]
