# Supplementary material for: Detecting Excess Biofilm Thickness in Microbial Electrolysis Cells by Real‐Time In‐Situ Biofilm Monitoring
Source: Biotechnol Bioeng. 2025 May 2;122(8):2049–62. doi: 10.1002/bit.29017 (PMC12235218; doi:10.1002/bit.29017)
Supplement: Supplementary file 10 — Table SI 1. [file BIT-122-2049-s006.docx]

Table SI-1: Comparison of the specific volumetric current production i_F_ of the electroactive biofilm during the growth in batch mode (Day 0-7) and during the continuous mode for Day 7 to the maximum current production

|  | Batch | | Continuous | |
| --- | --- | --- | --- | --- |
|  | i_F_ (A/mm³) | R² | i_F_ (A/mm³) | R² |
| MEC A1 | 55.456 | 0.95 | 18.23 | 0.67 |
| MEC A2 | 86.881 | 0.91 | 12.18 | 0.59 |
| MEC A3 | 78.113 | 0.88 | 26.22 | 0.86 |
| MEC B1 | 102.712 | 0.93 | 21.8 | 0.96 |
| MEC B2 | 162.217 | 0.97 | 53.02 | 0.96 |
| Mean | 97.076 ± 32.84 |  | 26.29 ± 12.902 |  |
